# Psychosocial factors as predictors of dropout in ultra-trailers

**DOI:** 10.1371/journal.pone.0206498

**Published:** 2018-11-05

**Authors:** Karine Corrion, Valérie Morales, Alessandro Bergamaschi, Bernard Massiera, Jean-Benoit Morin, Fabienne d’Arripe-Longueville

**Affiliations:** 1 Université Côte d’Azur, LAMHESS, Nice, France; 2 Université d’Orléans, ERCAE, Orléans, France; 3 Université Côte d'Azur, URMIS, Nice, France; University of Central Lancashire, UNITED KINGDOM

## Abstract

**Objective:**

Although research on the physiological and psychological factors of endurance performance has been extensive, the factors related to dropping out of ultra-trail races have not been well documented. The aim of this study was to examine psychosocial factors as predictors of dropout in ultra-trail runners.

**Methods:**

Two hundred and twenty-one volunteer athletes completed a survey that included measures of: (a) motivational variables (self-determined motivation, basic needs satisfaction, achievement goals), (b) theory of planned behavior constructs (attitudes, subjective norms, self-efficacy and intention to finish the race), and (c) coping strategies in sport.

**Results:**

The results showed that finishers had higher scores for self-efficacy and intention to finish the race than withdrawers, whereas withdrawers had higher scores for avoidance coping. Multiple logistic regression showed that the number of started and finished ultra-trail races (OR, 0.44; 95% CI 0.22–0.88; *p*<0.02), self-efficacy (OR, 2.03; 95% CI 1.06–3.89; *p*<0.04), intention to finish the race (OR, 0.34; 95% CI 0.16–0.71; *p*<0.004), mastery-approach goals (OR, 0.56; 95% CI 0.31–1.00; *p*<0.05), and coping strategies of seeking social support (OR, 0.43; 95% CI 0.26–0.71; *p*<0.001) were associated with a lower risk of race dropout, whereas avoidance coping strategies (OR, 2.26; 95% CI 1.46–3.52; *p*<0.001) were associated with an increased dropout rate.

**Conclusion:**

Interventions promoting self-efficacy constructs and specific coping strategies might contribute to preventing dropout in ultra-trailers.

## Introduction

In the last few decades, ultra-trail races have become very attractive for a wide range of athletes, from elite athletes to recreational runners, and participation in the popular ultra-races has grown remarkably [1]. An ultra-trail race is defined as any running race in a natural environment along a marked trail (ITRA; International Trail Running Association, 2013) longer than the classic trail distance of 42 kilometers. Due to a typical duration of more than 6 hours, it is considered an ultra-endurance sports event [2] and athletes must therefore push their bodies beyond the ordinary limits [3]. Moreover, as long (>80 km) and very-long (>160 km) ultra-trail races have become more popular [1, 4, 5], the participation of master athletes has notably increased [6, 7]. Indeed, recent studies have demonstrated that the age of peak running speed for ultra-marathoners tends to be above 35 years [8, 9, 10].

In addition to age considerations, ultra-endurance performance requires both physical and mental abilities [11], especially in ultra-trailing, where the dropout rate can reach 50% depending on the race (e.g., “Ultra-Trail du Mont-Blanc” [12], “Grand Raid de la Réunion” [13]). Over the years, researchers have been encouraged to describe the factors that promote human performance during prolonged endurance exercise. The evidence suggests that ultra-endurance athletes who compete in events lasting up to several days must cope with a number of acute physiological and psychological factors [14, 15, 16, 17]. For example, ultra-endurance athletes routinely must face such challenges as insufficient energy intake [18, 19], dehydration [20], elevated biomarkers of sympathetic stress [21] and cardiac damage [22], sleep deprivation [18, 23, 24], the accompanying alterations in emotional states [25], and intense unwanted emotions [16, 26].

The recent research on endurance sports (marathon, trail, triathlon) has mainly focused on the physiological factors of performance [27, 28], with most studies reporting on the key physiological variables that contribute to endurance performance, like anaerobic threshold, maximal oxygen uptake, economy of motion and the fractional utilization of oxygen uptake (e.g., [12, 29]). However, performance in ultra-endurance events, which are far longer than typical endurance events like the marathon, is much harder to predict based on the above-mentioned standard physiological features [28]. Ultra-trail running performance depends on several factors other than the energetic demand-supply or the energetic cost of running [30]. Holt et al. [27] showed that runners experienced numerous stressors (i.e., cramping and injuries, gastrointestinal problems, thoughts about quitting) during ultra-trail races. Also, ultra-marathon runners were found to have a higher proportion of stress fractures involving the foot among their annual incidence of exercise-related injuries [31]. Yet although the physiological features of long-distance runners have been extensively investigated, their psychological characteristics or strategies have been less well documented.

The early studies in the sports psychology literature investigated the cognitive strategies and motivation of runners [32, 33, 34, 35, 36, 37]. These works highlighted coping strategies like setting small goals, engaging in mental/physical battles, monitoring pace, and ensuring optimal nutrition, hydration, and social support. Ogles et al. [35] reported that external imagery was more effective for long-distance runners, who tended to dissociate from physical pain. Their motivations for participating in these events were various (e.g., making sense of one’s life, social recognition, achieving personal goals).

Recently, McCormick, Meijen and Marcora [38] conducted a systematic literature review to identify the psychological determinants of endurance performance. Their review indicated that nonrelevant imagery (e.g., [39]), lack of self-efficacy (e.g., [40, 41]), and mental fatigue (e.g., [42, 43]) were able to undermine endurance performance and that self-determined motivation did not have an impact on performance (e.g., [44]). In contrast, verbal encouragement and head-to-head competition (e.g., [45]), high self-efficacy and performance orientation (e.g., [40, 46, 47]), and relevant goal setting (e.g., [41, 48, 49]) had beneficial effects and improved performance. Mental strategies (including pre-competition routines, pre-race use of energizing strategies, and setting outcome goals for the race), competitive motivation for participating [50], motivational self-talk [51] and stress and recovery states [52] were also found to improve end performance.

A few studies have focused on the role of motivation and attitudes in race dropout (e.g., [53], and others have examined the constructs underpinning the theory of planned behavior (TPB) and the key motivational factors involved in an athlete’s intention to participate in future sports or ultra-endurance events (e.g., [54]). In a meta-analytic review, Armitage and Conner [55] found that TPB accounted for more than 11% of the variance in behavior. TPB puts the emphasis on perceived subjective norms and personal attitudes about engaging in a behavior as predictors of the intention to engage in the behavior [56]. In this regard, subjective norms are perceptions about whether or not a behavior will be approved of by a relevant reference group. Another relevant construct is Bandura’s [57] self-efficacy, which is defined as the perceived difficulty of successfully carrying out a behavior that can be influenced by past experience, expected support, modeling, and potential obstacles.

Given the high dropout rate in ultra-endurance sports, a related issue worthy of exploration is how athletes persevere and cope with discomfort and pain. Previous studies have reported that high-level athletes use effective, specific cognitive strategies such as social support and active thoughts [27, 58, 59, 60]. Baker and Sedgwick [61] showed that expert triathletes had different cognitive characteristics from their less experienced counterparts. Experts reported a greater emphasis on thoughts related to their performance and were more proactive in their approach to performance situations, while middle of the pack and back of the pack triathletes reported a greater number of passive thoughts. Furthermore, Simpson, Post, Young and Jensen [62] indicated that preparation and strategy (i.e., physical training, nutrition) were among the major themes characterizing the experience of ultra-marathon running. Overall, these results suggest that optimal training versus under-preparation is at the heart of most decisions to dropout because of both physiological barriers like fatigue and injury and psychological barriers like inadequate strategies and coping skills.

Despite the high dropout rate in ultra-trail races (i.e., sometimes more than 50%), which may have negative personal, organizational and economic consequences, research into the reasons for dropout have been sparse. Two recent qualitative studies nevertheless focused on this topic [13, 63]. Antonini et al. [63] showed that during runners’ courses of experience, a complex set of factors including the experience of pain, attempts to overcome problems, assessments of the current situation, and disruptive events inevitably led to withdrawal. Rochat et al. [13] showed that race finishers and withdrawers differed in the organization of their vitality adaptations and the way they dealt with difficulties. Specifically, finishers completed the race with higher states of vitality preservation and lower states of vitality loss than withdrawers. Although this study provided the first data on the psychological states associated with dropping out of ultra-trail races, the role of many of the psychosocial factors related to motivation and coping remains unexplored.

The purpose of the present study was to further examine the role of psychosocial factors as predictors of dropout in ultra-trailers. Previous studies on sports participation and dropout have consistently reported that sociocognitive constructs like achievement motivation and attitudes (e.g., [53]) and coping strategies are predominant in endurance performance (e.g., [13, 38]), and these variables were therefore retained as potential variables to explain dropout. Specifically, we expected that achievement motivation characteristics (i.e., self-determined motivation, basic needs satisfaction, and achievement goals), constructs derived from TPB (attitudes, subjective norms, self-efficacy and intention to finish the race), and coping strategies would be significant predictors of dropout.

## Materials and methods

### Participants and sampling procedure

The sample was composed of 221 volunteer athletes from 18 to 61 years old *(M*_*age*_ = 43; *SD* = 5.56), with195 males (*M*_*age*_ = 42; *SD* = 5.55) and 26 females (*M*_*age*_ = 45; *SD* = 11.24). They were predominantly from the upper class (see Table 1) and all participated in the “Côte d’Azur Mercantour Ultra-Trail” (140 km; 10,000 m of positive elevation). A total of 519 athletes took part in this event, with 262 finishers and 257 non-finishers. In our sample, 96 athletes finished the race and 125 did not. The participants presented a wide range of ultra-trail (UT) experience and training volumes (Table 1). All participants signed a consent form and were free to withdraw at any point.

**Table 1:** Characteristics of participating athletes (N = 221).

	Total (n = 221)	Finishers (n = 96)	Non-finishers (n = 125)
**Socio-professional categories**			
Upper class	51.3%	55 (24.9%)	66 (29.8%)
Middle class	33.0%	29 (13.1%)	41 (18.6%)
Lower class	11.6%	12 (5.4%)	18 (8.1%)
**Males**, n (%)	195 (88.2%)	85 (38.4%)	110 (49.8%)
**Females**, n (%)	26 (11.8%)	11 (5.0%)	15 (6.8%)
**Expert**	94 (42.5%)	54 (24.4%)	40 (18.1%)
(Finished more than 3 races)			
*M* years of experience	9.52	9.30	9.82
*M* finished-race experience (numbers of UTs)	10.08	10.59	9.4
*M* weekly training volume (hours)	8.54	11.5	8.96
**Novices**	127 (57.5%)	44 (19.9%)	83 (37.6%)
(0 to 3 races finished)			
*M* years of experience	4.79	5.23	4.56
*M* finished-race experience (numbers of UTs)	1.08	1.61	0.80
*M* weekly training volume (hours)	9.46	10.88	8.12

### Procedure

The principal investigators obtained approval for this project from the human ethics committee of Nice Sophia-Antipolis University prior to starting the study. The questionnaires were completed before the race began (at the starting site, one day before the official start of the race). All 519 registered athletes were invited to participate. Standardized information and instructions for the questionnaires were given to the athletes to ensure optimal conditions and attentiveness on their part. We provided the following information: (a) participation in the study was strictly voluntary, (b) the questionnaires were not tests and therefore there were no right or wrong answers, and (c) the collected data would be used only for research and would remain strictly confidential. All questionnaire sessions were held in standardized conditions (i.e., small groups, paper, pencils, seating and no communication) or online. The sessions lasted 20 minutes at most. The status of finisher or withdrawer was matched to each participant’s registration number.

### Measures

The survey was composed of two sections. The athletes first provided demographic information and answered a series of questions pertaining to their experience with trail and UT running (i.e., number of UTs, number of started and finished UTs, years of practice, average training time per week). They then completed a series of questionnaires to measure the following variables: (a) motivational variables (self-determined motivation, basic needs satisfaction, achievement goals), (b) TPB constructs (attitudes and subjective norms related to UTs, self-efficacy related to the race and intention to finish the race), and (c) coping strategies in UT races. For all these questionnaires, participants were invited to recall what they usually did and thought about while UT running and during past UT races, and to complete each item accordingly.

#### Motivation for UT

Motivation for UT was measured using an adapted version of the French Sport Motivation Scale II (SMS-II). This scale is an 18-item questionnaire that measures the six motivation orientations described by self-determination theory (SDT) in the sports context [64] on a 5-point Likert-type scale, with responses ranging from 1: *Strongly disagree* to 5: *Strongly agree*. The term “sport” was replaced by “ultra-trail” for all items. As the participants were all athletes, the amotivation subscale was not considered. A CFA showed that the model was significantly adjusted to the data [i.e., χ*^2^* (125) = 235.47; *N* = 221; CFI = 0.91; TLI = 0.90; RMSEA = 0.063; CI RMSEA = 0.051/0.076]. The scale demonstrated satisfactory internal consistency (0.74 for intrinsic motivation and 0.78 for extrinsic motivation).

#### Basic psychological needs

Basic psychological needs satisfaction was measured using the 15-item scale developed by Gillet et al. [65]. This scale is composed to three subscales with five items: (a) competence (e.g., “I feel successful as an ultra-trailer”), (b) autonomy (e.g., “As an ultra-trailer, I feel free to make my own choices”), and (c) affiliation (e.g., “As an ultra-trailer, I have a feeling of closeness with the other runners I interact with”) on a Likert scale from 1: *Strongly disagree* to 5: *Strongly agree*. In this study, the Cronbach alpha was 0.73.

#### Achievement goals

The French Achievement Goals Questionnaire for Sport and Exercise (FAGQSE; [66]) was used to assess the four goals in the 2 X 2 model (i.e., mastery-approach, performance approach, mastery-avoidance, performance-avoidance). Each item began as follows: “When I am ultra-trail running ….” Three items assessed each goal (mastery-approach: e.g., “I want to improve”; performance-approach: e.g., “I want to do better than the others”; mastery-avoidance: e.g., “I want to avoid technically declining”; and performance-avoidance: e.g., “I want to avoid performing worse than the others”). Participants responded on a scale from 1: *Strongly disagree* to 5: *Strongly agree*, and average scores were computed for each achievement goal variable. The alphas of the mastery-approach, performance-approach, mastery-avoidance, and performance-avoidance goals were, respectively, 0.87, 0.90, 0.88 and 0.91.

#### Theory of planned behavior (TPB) constructs

The variables derived from TPB (attitudes, subjective norms, self-efficacy and intentions) were measured using the guidelines provided by Ajzen [56]. Attitudes were measured with the stem proposition “Your attitude about participating in UTs is…” followed by two semantic differential evaluative adjectives (useless/useful; harmful/beneficial) scored on a 5-point scale. Subjective norms were assessed with the mean of three items (e.g., “Most people who are important to me would want me to participate in a UT”) scored on a 5-point scale: 1: *Strongly disagree* to 5: *Strongly agree*. Intention was measured with the mean of two items (e.g., “I intend to finish the race”) scored on 5-point Likert scale (1: *Definitely not* to 5: *Definitely yes*). Self-efficacy was assessed with the mean of three items (e.g., “I feel able to go all the way”) on a 5-point Likert-type scale with responses ranging from 1: *Strongly disagree* to 5: *Strongly agree*. The internal consistency of the scale was satisfactory (α = 0.75).

#### Coping strategies in UT

Coping strategies in UT were evaluated with the French version of the brief COPE Inventory [67, 68]. The questionnaire is composed of 18 items related to four subscales: (a) seeking social support (i.e., venting, emotional and instrumental support, and religion), (b) problem solving (i.e., active coping and planning), (c) avoidance (i.e., behavioral disengagement, self-distraction, substance use, denial, and self-blame), and (d) positive thinking (i.e., humor, positive reframing, and acceptance). All items of the scale are rated on a 5-point Likert-type scale, ranging from 1: *Not at all* to 5: *Very much so* (5). Given the specific context of UTs, a few irrelevant items (i.e., related to religion or humor) were not considered. The CFA showed that the four-item model was significantly adjusted to the data [i.e., χ*^2^* (126) = 222.56; *N* = 221; CFI = 0.97; TLI = 0.94; RMSEA = 0.070; CI RMSEA = 0.033/0.106], and the scale demonstrated satisfactory internal consistency (α = 0.85).

### Data analyses

All statistical analyses were performed using SPSS.22 [69]. The analysis authorized the replacement of missing values by multiple imputation [70]. We began with descriptive analyses (i.e., means, standard deviations) and assessed the reliability of the questionnaires with Cronbach’s alpha for internal consistency. Pearson bivariate correlations were computed to assess the significance of the relationships between all variables. We next examined the differences between finishers and withdrawers on all the measures using a multivariate analysis of variance (MANOVA). Then, we performed multiple logistic regression analyses to identify the contribution of each type of psychosocial variable to dropout. The odds ratios (OR) with 95% confidence intervals (CI) were used as a measure of the strength of the association between the presence of variables and the occurrence of an event.

## Results

Descriptive statistics and correlations among the variables are presented in Tables 1, 2 and 3. Overall, the study sample was characterized by a high proportion of men (88.2%) and 51.3% belonged to the upper class; 42.5% were UT experts and 57.5% were novices.

**Table 2:** Descriptive statistics.

	Total population (n = 221)		Finishers (n = 96)		Non-finishers (n = 125)	
	*M*	*SD*	*M*	*SD*	*M*	*SD*
**Motivation**						
Intrinsic motivation	3.25	0.96	3.27	0.96	3.23	0.97
Extrinsic motivation	2.49	0.60	2.50	0.62	2.47	0.58
**TPB constructs**						
Attitude	3.01	0.84	3.12	0.77	2.92	0.88
Subjective norms	1.50	0.76	1.56	0.82	1.44	0.72
Intention	4.09	0.74	4.34*	0.55	3.07	0.88
Self-efficacy	4.03	0.83	4.22*	0.69	3.87	0.96
**Coping Strategies**						
Seeking social support	3.68	0.80	3.79	0.80	3.61	0.81
Problem solving	3.64	0.80	3.56	0.85	3.70	0.75
Avoidance	3.24	0.68	2.59	0.88	2.99*	0.89
Positive thinking	3.17	0.77	3.15	0.71	3.19	0.79
**Basic Psychological Needs**						
Competence	2.78	0.51	2.72	0.49	2.83	0.53
Autonomy	4.03	0.71	4.15	0.71	3.94	0.70
Affiliation	3.86	0.71	3.95	0.67	3.79	0.73
**Achievement Goals**						
Mastery-approach	3.95	0.79	4.07	0.80	3.86	0.77
Mastery-avoidance	2.02	1.04	2.02	1.06	2.02	1.03
Performance approach	3.61	0.82	3.71	0.84	3.53	0.80
Performance avoidance	2.31	1.07	2.14	1.13	2.12	1.03
**Expertise**						
Years of experience	6.80	8.77	7.47	4.95	6.26	5.15
Weekly training volume	5.10	7.44	9.64	10.15	8.14	4.41

*Notes*. *M*: Means; *SD*: Standard Deviations; TPB: Theory of Planned Behavior

**Table 3:** Matrix of factor correlations (N = 221).

	IM	EM	ATT	SN	INT	SE	Cop	NS	MG	PG
Intrinsic motivation	-									
Extrinsic motivation	0.58**	-								
Attitude	0.39**	0.56**	-							
Subjective norms	0.13	0.21**	0.14*	-						
Intention	0.21**	0.21**	0.23**	0.01	-					
Self-efficacy	0.35**	0.28**	0.31**	0.13	0.74**	-				
Coping strategies	0.30**	0.34**	0.24**	0.05	0.13	0.18**	-			
Needs satisfaction	0.23**	0.25**	0.15*	0.07	0.30**	0.29**	0.28**	-		
Mastery goals	0.36**	0.30**	0.33**	0.04	0.29**	0.37**	0.28**	0.49**	-	
Performance goals	0.13*	0.16*	0.09	0.19*	0.17*	0.19**	0.22**	0.02	0.34**	-

*Notes*. IM: Intrinsic motivation; EM: Extrinsic motivation; ATT: Attitude; SN: Subjective norms; INT: Intention

SE: Self-efficacy; Cop: Coping strategies; NS: Needs satisfaction; MG: Mastery goals; PG: Performance goals

*p < .05

**p < .01; 0.1–0.3: small; 0.3–0.5; moderate; 0.5–0.7; large; 0.7–0.9: very large.

Examination of the means of the variables showed that experts scored moderate to high on years of experience and weekly training volume. In addition, the participants scored high on intention, self-efficacy, autonomy, mastery and performance approach goals, and low on subjective norms. Pearson’s correlations showed that (a) intrinsic and extrinsic motivation were significantly related to attitude, intention, self-efficacy, coping strategies, needs satisfaction and mastery goals; (b) intention was significantly linked to self-efficacy, needs satisfaction and mastery goals, and (c) self-efficacy and coping strategies were significantly related to needs satisfaction and achievement goals.

### Differences between finishers and non-finishers

The MANOVA performed on all variables yielded significant differences between the two groups [Wilk’s λ = 0.86, *F*(3,217) = 11.499, *p*<0.001, η^2^ = 0.14]. Subsequent ANOVAs showed significant differences between finishers and non-finishers on (a) UT self-efficacy, *F*(1, 219) = 9.168, *p*<0.01, η^2^ = 0.025, with the finishers scoring significantly higher on self-efficacy before the race (*M*_*F*_ = 4.22, *SD* = 0.69) than the non-finishers (*M*_*W*_ = 3.87, *SD* = 0.96); (b) intention to finish the race, *F*(1, 219) = 20.685, *p*<0.001, η^2^ = 0.061, with the finishers scoring significantly higher on intention to finish the race (*M*_*F*_ = 4.34, *SD* = 0.55) than the non-finishers (*M*_*W*_ = 3.07, *SD* = 0.88); and (c) avoidance coping strategies, *F*(1, 219) = 14.996, *p*<0.001, η^2^ = 0.064, with the non-finishers adopting significantly more avoidance coping strategies (*M*_*W*_ = 2.99, *SD* = 0.89) than the finishers (*M*_*F*_ = 2.59, *SD* = 0.88). Other variables did not significantly differ between the groups (i.e., gender, motivation, basic psychological needs, attitudes, subjective norms, achievement goals, and other coping strategies).

### Psychosocial variables as predictors of dropout

The model of multiple logistic regression explained 36.4% of the variance in UT dropout. The results showed that a high number of started and finished UTs (OR, 0.44; 95% CI 0.22–0.88; *p*<0.02), high self-efficacy (OR, 2.03; 95% CI 1.06–3.89; *p*<0.04), strong intention to finish the race (OR, 0.34; 95% CI 0.16–0.71; *p*<0.004), mastery-approach goals (OR, 0.56; 95% CI 0.31–1.00; *p*<0.05), and coping strategies of seeking social support (OR, 0.43; 95% CI 0.26–0.71; *p*<0.001) were associated with a lower risk of dropout. In contrast, avoidance coping strategies (OR, 2.26; 95% CI 1.46–3.52; *p*<0.001) were associated with a higher risk of dropout.

## Discussion

The aim of this study was to examine psychosocial factors as predictors of dropout in ultra-trailers. We expected that motivation, coping strategies, TPB variables (attitudes, subjective norms, self-efficacy and intention) and basic needs satisfaction would be associated with dropout in the UT competitors.

First, our results show differences between finishers and non-finishers on several psychological variables of interest. The finishers scored significantly higher on self-efficacy and intention to finish the race than the non-finishers, these latter having reported higher scores of avoidance coping strategies. Furthermore, self-efficacy, intention, mastery-approach goals and seeking social support to finish the race were associated with a lower risk of dropout. However, it should be acknowledged that the size of these effects was small. This set of findings is in line with previous studies showing the positive role of self-perceptions in endurance sports of shorter duration (e.g., [40, 41]. They extend the adaptive role of the self-efficacy theory constructs evidenced in many sports performances [71] to the context of UT races. They are also consistent with the recent data from Rochat et al. [13] showing the role of preservation of vitality in UT finishers.

Second, the findings of the present research provide insight into the sociodemographic characteristics of the non-finishers, who were predominantly male and of high socioeconomic status, similarly to the finishers. These results are in line with previous studies reporting that ultra-trailers generally tend to have high socioeconomic status and to be well-educated [72] and predominantly male (e.g., [73, 74]). From a descriptive point of view, the socioeconomic status does not seem to play a role in dropout. Furthermore, and although the number of females of our sample was small, our results did not show any differences between males and females on psychosocial variables.

Third, the results of the multiple logistic regression analysis show that a low number of started and finished UTs, avoidance coping self-efficacy and low scores for intention, mastery-approach goals and seeking social support to finish the race were associated with increased dropout in the ultra-trailers. Consistent with the research showing the role of experience in ultra-marathon running (e.g., [61, 62]), the contribution of the number of started and finished UTs in explaining dropout suggests the importance of athletes being prepared and feeling confident based on past experiences [57].

Furthermore, in line with previous studies [27, 58, 59, 60], our results show that runners who finish the race use specific coping strategies, notably seeking support. Interestingly, our findings also indicate that avoidance coping strategies increase the risk of dropout and can thus be considered as risk factors. This study is the first to provide evidence that these coping strategies may predict UT dropout, thus extending the maladaptive consequences associated with avoidance coping strategies reported in the sports psychology literature (e.g., [75]). It also provides a deeper understanding of the cognitive strategies involved in UT races and complements previous studies that have examined the role of cognitive orientation during competition [35, 58, 76] and mental toughness in ultra-endurance competition [21, 63].

Contrary to our hypotheses, neither self-determined motivation for UT nor needs satisfaction was associated with dropout in our study. In their systematic review, McCormick et al. [38] reported that psychological interventions aimed at increasing motivation (e.g., through statements, self-talk or priming for an autonomous motivation orientation) contributed to endurance performance. This discrepancy with the literature might be due to the different measures of motivation. Whereas previous studies considered motivational states through psychological interventions (e.g., [40, 50]), the present research focused on more dispositional motivational variables. From this perspective, although the characteristics of the finishers and non-finishers of the present study were not significantly different, finishers’ scores for autonomy, mastery-approach goals and performance-approach goals were slightly higher (diff. in means: 0.2) at the descriptive level. In addition, mastery-approach goals were associated with a lower risk of dropout. To our knowledge, this is an original finding in the ultra-endurance literature, extending the adaptive consequences of the achievement goals already outlined in other sports and academic contexts (e.g., [77, 78, 79]).

Several potential limitations of this study should be considered when interpreting these findings. First, the data for our quite sensitive variables were self-reported and may thus have been subject to social desirability bias. Second, the generalizability of the results is limited by the nature and type of the ultra-trail race, which was a first edition, and the characteristics of the sample. This study could thus be replicated in other samples and UT races to strengthen the generalizability of the findings.

### Perspectives

This study provides original findings on the psychosocial variables that contribute to dropout in ultra-trail races. Yet it also suggests the interest of exploring the roles of other psychological and physiological variables (e.g., personality traits, physical preparation, perceived and neuromuscular fatigue) and their relative contributions to ultra-performances. Furthermore, as the training volume of the finishers and non-finishers was quite similar in the present research, and as previous positive experience is known to be the major source of self-efficacy [57], future work could try to better identify the role of expertise.

The present study has interesting practical implications. Our findings suggest that effective interventions should try to promote self-efficacy, mastery-approach goals, and the coping strategy of seeking social support to better empower athletes and their entourage to avoid dropout. This underlines the need to educate coaches and athletes regarding the psychological skills most likely to reduce the risk of dropout.

## Conclusions

Our results showed that self-efficacy, mastery-approach goals, intention to finish the race, and seeking social support protected against dropout in ultra-trail races, whereas avoidance coping was a risk factor of dropout. Interventions promoting self-efficacy, self-referenced goals and effective coping strategies might contribute to preventing dropout in ultra-trailers.

## Supporting information

S1 File IM: Intrinsic motivation; EM: Extrinsic motivation; ATT: Attitude; SN: Subjective norms; INT: Intention; SE: Self-efficacy; Cop: Coping strategies; NS: Needs satisfaction; MG: Mastery goals; PG: Performance goals. (PDF)
