# Supplementary material for: Psychosocial factors as predictors of dropout in ultra-trailers
Source: PLoS One. 2018 Nov 5;13(11):e0206498. doi: 10.1371/journal.pone.0206498 (PMC6218041; doi:10.1371/journal.pone.0206498)
Supplement: S1 File — (PDF) [file pone.0206498.s001.pdf]

									Motivation																			
Sexe 1=F /2=	Date of birthday	0 = dropout/ 1 =	Years of practice	Average training time per	Level	strategies of food	Number of started UTs	Number of finished UTs	EM1	AM1	IM1	EM2	EM3	EM4	EM5	EM6	IM2	AM2	EM7	EM8	AM3	EM9	EM10	EM11	IM3	EM12	SN1	ATT1
2	1983	1	1	10	4	4	0	0	3	1	2	2	1	4	3	1	2	1	3	2	3	2	3	5	4	4	1	2
2	1960	0	6	15	4	4	5	5	5	1	5	5	1	4	1	1	5	1	4	4	1	4	1	5	4	4	4	5
2	1992	0	0	15	3	5	0	0	1	1	4	4	1	5	1	1	4	1	1	3	1	4	1	2	4	4	1	2
2	1989	0	1	7	3	3	0	0	1	1	3	5	1	4	1	1	2	1	2	4	1	4	1	3	3	4	2	3
2	1962	0	3	5	3	2	0	0	1	1	1	2	1	3	1	1	2	1	1	3	1	2	1	1	4	4	1	2
2	1981	0	1	5	3	4	1	1	1	4	5	5	1	5	1	1	3	5	5	4	3	5	1	5	5	5	1	4
1	1979	0	2	5	3	3	0	0	3	1	4	5	1	2	1	1	2	1	2	2	1	4	1	3	5	5	1	4
2	1981	0	1,5	8	3	3	2	1	1	1	4	2	1	4	1	1	3	1	1	2	1	4	1	3	4	4	1	2
2	1972	1	3	7	3	2	0	0	3	3	2	2	1	2	1	1	3	1	3	2	1	1	1	1	2	2	1	4
2	1972	0	3	15	3	4	0	0	4	1	3	5	3	5	1	1	4	1	4	4	1	4	3	4	4	5	2	5
2	1964	1	6	10	3	4	0	3	1	1	2	4	1	3	1	1	4	1	3	3	3	2	1	2	2	2	4	4
2	1952	1	11	8	3	4	8	6	2	1	1	4	1	4	1	1	1	1	2	2	1	4	1	4	2	2	1	2
2	1986	1	2	14	3	5	0	0	1	1	4	4	1	4	1	1	4	1	5	5	1	4	1	4	5	4	1	3
2	1973	1	8	5	5	4	1	1	1	1	1	4	1	4	4	3	4	1	4	5	1	4	1	5	4	4	1	2
2	1967	0	1	44	1	5	0	0	5	1	3	3	1	3						3	1	3	1	3	5	4	1	5
2	1969	0	5	15	4	3	3	2	5	1	2	2	1	3	1	1	1	1	2	3	1	2	1	2	2	2	1	2
2	1981	1	5	15	2	2	5	4	1	1	4	5	1	5	1	1	4	1	3	4	1	4	1	5	4	5	1	4
2	1965	1	10	10	3	4	18	15	4	1	4	5	1	5	1	1	4	1	5	4	1	5	1	4	4	4	2	4

2	1963	0	2	5	4	3	2	1	1	2	2	4	1	2	1	1	2	2	2	2	1	1	1	4	1	1	1	2
1	1978	0	10	5	2	5	4	3	4	1	2	5	1	5	3	1	5	5	5	5	1	5	1	5	5	5	1	5
2	1966	1	5	11	3	3	3	2	3	1	2	2	1	2	1	1	3	1	2	2	1	2	1	2	2	2	2	2
2	1973	0	15	9	2	5	8	8	5	1	4	5	3	4	1	1	4	1	4	1	1	5	1	5	4	4	1	4
2	1958	0	8	24	3	2	1	1	3	2	4	4	1	4	1	1	4	3	1	4	1	3	1	4	4	4	4	4
2	1973	0	5	11	3	4	4	3	2	1	5	4	3	4	1	1	4	1	3	3	1	4	1	2	4	4	4	4
2	1964	1	30	9	3	3	10	8	2	1	4	4	1	4	1	1	4	1	4	4	1	4	1	4	4	4	1	4
2	1963	0	2	27	3	3	0	0	3	3	3	3	3	3	3	3	2	1	2	3	1	2	1	3	2	2	1	3
1	1958	0	10	7	3	4	9	9	1	1	4	5	1	5	1	1	5	1	5	4	1	4	1	4	4	5	1	4
2	1962	0	10	5	4	3	10	10	4	1	3	3	1	2	2	1	2	1	3	2	1	2	1	3	2	3	1	2
2	1979	1	10	6	4	3	2	2	3	1	3	5	1	5	3	1	1	1	2	4	1	3	1	4	3	5	1	4
2	1979	0	5	4	3	1	2	1	2	1	4	5	3	4	3	1	4	1	3	4	1	4	3	5	5	4	2	3
2	1969	0	15	5	3	4	0	0	3	1	2	1	1	2	3	1	3	1	2	2	1	2	3	2	2	2	5	3
2	1965	0	4	8	3	4	40	30	4	1	4	4	1	4	1	1	4	1	4	4	3	4	2	4	4	4	1	4
2	1973	0	3	15	4	3	1	1	4	1	4	4	1	4	1	1	4	1	4	3	1	4	4	5	4	5	1	4
2	1981	1	7	15	3	1	8	7	5	1	3	3	1	3	1	3	3	1	3	3	1	3	1	4	3	3	3	3
2	1969	1	5,5	7	3	3	12	10	3	1	4	4	1	2	1	1	4	1	3	1	1	4	1	3	4	1	1	4
2	1972	1	8	6	2	2	2	1	1	3	3	4	1	4	1	1	4	1	1	3	3	1	1	2	3	3	1	2
2	1974	0	2	10	3	3	0	0	1	1	5	5	1	5	1	1	5	1	5	1	5	1	1	1	5	5	1	3

2	1955	1	20	8	3	4	15	8	4	1	4	3	1	4	1	1	4	1	2	3	1	4	1	4	4	3	1	5
2	1964	1	10	3	4	3	15	14	3	1	4	5	1	5	1	1	4	1	4	4	1	4	1	4	4	5	4	4
2	1974	1	12	4	3	4	6	5	1	1	2	2	1	2	1	1	4	1	4	3	1	4	1	5	4	3	3	3
2	1979	0	8	4	4	3	4	4	1	1	3	3	1	1	1	1	3	1	3	3	1	3	3	4	3	3	1	3
2	1970	1	5	10	3	3	10	10	2	1	2	2	1	5	1	1	2	1	4	5	1	5	1	4	4	4	3	4
2	1976	0	5	4	4	4	4	3	3	1	4	4	1	1	1	1	4	1	1	1	1	2	1	2	3	2	2	1
2	1977	0	10	4	2	3	1	0	1	3	5	3	3	3	1	1	2	2	1	1	3	1	1	1	2	1	1	1
2	1981	1	5	12	3	3	3	2	4	1	4	2	2	2	2	1	4	1	2	2	1	4	1	4	4	2	3	4
2	1985	0	1	12	3	2	0	0	1	1	5	4	1	5	1	1	5	1	1	4	5	5	1	1	4	5	1	4
2	1974	1	2	10	4	4	4	4	1	1	1	5	1	2	1	1	1	1	2	2	1	5	1	1	2	2	1	3
2	1973	1	10	10	5	4	20	18	3	1	3	2	3	5	2	3	5	1	5	5	1	5	3	5	5	5	2	5
2	1978	0	6	18	3	3	5	4	1	1	2	2	1	4	1	1	2	1	2	2	1	4	1	2	1	2	1	1
2	1980	0	4	6	3	4	1	1	1	1	2	4	1	4	1	1	2	1	2	4	1	4	1	5	5	4	1	4
2	1960	0	12	4	2	2	10	9	1	1	2	2	1	2	2	1	1	1	1	1	1	2	1	4	4	3	4	2
2	84	1	3	8	4	3	3	3	4	1	4	4	3	4	1	1	4	1	4	4	1	4	3	5	4	4	3	4
2	1978	1	1,5	10	4	4	3	3	1	1	4	5	1	4	1	1	5	1	5	1	1	5	5	5	5	5	5	5
2	1970	1	5	12	4	4	3	3	1	1	5	5	1	4	1	1	3	1	1	3	1	2	1	1	4	4	1	4
2	1961	1	6	17	3	3	3	3	1	3	2	2	1	4	1	1	4	3	1	4	1	3	1	1	2	2	1	2
2	1977	1	10	8	4	3	55	55	3	1	3	3	1	4	1	1	3	1	3	4	1	3	4	3	4	4	4	3

2	1959	1	8	9	3	2	10	10	1	1	2	3	1	2	1	1	2	1	2	2	1	2	1	3	1	2	1	1
1	1983	0	4	8	4	4	1	0	1	1	3	4	1	2	1	1	2	1	3	4	1	2	1	1	2	4	1	4
2	1984	0	4	10	2	3	8	6	2	3	4	4	1	4	1	1	3	1	3	3	1	2	1	4	4	3	1	4
2	1968	0	1	2	3	2	1	1	1	3	4	3	1	2	1	1	2	1	1	2	1	2	3	3	1	2	1	2
2	1979	1	15	5	3	2	2	2	2	3	3	4	1	4	1	1	2	1	2	2	1	4	1	2	4	2	1	4
2	1971	1	8	15	2	1	3	3	3	1	4	4	1	2	2	1	3	1	3	2	3	2	1	2	2	2	1	4
2	1970	1	10	6	4	2	9	8	3	1	1	2	1	1	1	1	2	1	4	1	3	1	1	4	2	1	3	2
2	1966	0	5	7	3	3	9	4	4	1	2	2	1	2	2	1	3	3	4	4	2	3	2	5	4	3	3	3
2	1981	1	5	8	5	4	3	3	3	1	4	4	1	4	1	1	4	1	2	3	1	3	1	4	4	4	1	4
2	1951	1	3	8	3	2	1	1	1	1	4	3	1	3	1	1	4	1	3	1	4	1	1	3	3	2	3	3
2	1992	1	1	3	4	4	0	0	1	1	4	5	1	4	3	1	4	1	5	5	1	5	2	4	5	4	4	4
2	1960	0	10	6	3	3	2	1	3	2	4	3	2	3	3	1	4	2	2	3	2	2	1	3	3	3	2	3
2	1964	1	16	5	3	1	15	13	3	1	3	5	2	3	1	1	2	3	2	1	1	4	1	2	3	3	2	2
2	1973	1	7	10	4	4	5	4	4	1	5	4	1	2	1	1	5	1	1	3	1	5	1	4	5	5	1	2
2	1980	0	3	11	4	1	5	5	3	2	1	5	1	4	3	1	1	3	2	2	3	4	1	4	4	2	1	2
1	1984	1	3	11	4	1	3	2	3	1	5	5	1	5	1	1	2	1	4	4	1	4	2	4	4	5	1	3
2	1972	0	1	7	3	2	1	1	1	1	3	3	1	2	1	1	1	1	2	1	3	2	1	1	4	2	1	2
2	1972	0	4	12	4	3	1	1	1	1	4	4	1	3	1	1	3	1	2	3	1	3	1	4	4	3	1	3
2	1947	0	6	15	3	2	5	2	3	2	2	4	3	4	4	3	3	1	4	4	1	2	3	4	4	4	2	4
2	1953	0	5		3	3	2	0	3	4	3	3	4	3						4	4	4	4	5	3	3	2	3

2	1962	0	2	15	3	3	0	0	2	1	3	2	1	4	1	1	3	1	3	4	1	4	1	4	2	3	1	4
1	1958	1	10	15	3	2	3	2	1	1	1	5	1	4	1	1	1	1	5	5	1	4	1	2	5	2	1	4
1	1972	1	10	12	3	1	5	5	4	1	3	4	1	4	1	4	4	4	4	4	1	2	1	5	5	4	1	5
2	1969	1	5	4	3	3	7	6	5	2	5	4	1	4	2	1	4	2	4	3	2	4	1	5	4	4	1	4
2	1981	0	4	3	3	4	8	8	5	4	4	3	1	2	1	1	2	1	1	1	1	1	1	2	4	4	1	1
2	1984	0	2	4	2	1	0	0	1	1	1	2	1	2	2	1	3	1	3	2	1	2	1	4	4	3	1	4
2	1982	1	8	8	4	3	10	9	3	1	2	3	1	2	1	1	2	1	3	3	1	2	1	2	2	2	1	3
2	1984	0	1	5	1	4	0	0	1	1	1	4	1	4	1	1	3		1	4	1	4	1	3	4	5	1	4
2	1988	1	2	10	4	2	10	10	1	1	5	4	1	5	1	1	5	1	5	5	1	5	1	5	5	5	1	5
2	1953	0	12	5	3	4	15	11	2	1	2	4	1	4	2	1	2	1	3	4	1	4	1	4	2	2	1	2
2	1978	1	7	5	4	3	4	4	3	1	1	5	1	4	1	1	3	1	3	1	1	4	1	2	4	3	1	4
2	1965	0	10	4	4	4	1	0	3	2	2	2	1	4	1	1	4	1	4	1	1	4	1	4	4	4	1	3
2	1986	1	6	11	4	2	15	13	1	1	4	5	1	3	1	2	4	1	5	3	1	3	1	4	4	4	3	5
2	1981	1	3	12	3	4	0	0	1	1	4	4	1	2	1	1	5	1	2	2	1	2	2	2	2	4	4	4
2	1981	1	7	6	3	1	4	4	1	1	3	2	1	3	1	1	1	1	3	2	1	2	1	4	4	3	1	3
1	1982	1	4	12	2	3	5	5	3	1	4	4	2	4	1	1	5	1	2	4	1	3	2	4	4	5	1	3
2	1956	0	18	4	2	2	5	2	3	1	1	3	1	2	1	1	1	1	2	2	1	1	1	3	1	2	1	2
2	1970	0	1	8	1	2	0	0	2	1	4	4	1	3	1	1	4	4	4	5	3	4	1	3	3	3	1	3
1	1972	1	4	6	2	2	0	0	3	3	3	3	1	3	1	1	2	1	3	2	1	4	1	1	2	4	1	4
2	1990	0	6	9	4	5	0	0	1	1	4	4	3	5	1	1	4	1	4	4	1	4	4	3	5	4	4	4
2	1988	1	1	6	3	4	0	0	1	1	2	2	1	3	3	1	2	1	3	2	1	4	1	5	4	4	3	4
2	1962	1	12	5	3	2	6	6	1	1	2	2	1	2	1	1	2	1	1	2	1	2	2	1	2	2	1	2

2	1955	0	3	8	3	3	1	1	1	1	4	2	3	2	1	1	2	1	2	2	1	2	1	2	2	5	1	2
2	1968	0	10	15	4	4	2	2	1	1	4	4	1	4	1	1	4	1	3	2	1	1	1	2	4	4	1	4
2	1972	0	3	13	4	2	1	1	5	2	4	5	1	5	3	2	5	1	4	5	1	5	2	5	5	4	1	5
2	1981	1	4	12	5	5	2	2	3	1	2	2	1	4	1	1	2	1	2	2	1	2	1	3	2	3	1	3
2	1962	0	7	8	3	3	4	1	2	5	2	2	1	3	1	1	3	3	3	1	1	2	1	2	2	2	1	2
2	1973	1	5	10	4	5	1	1	1	1	3	3	1	3	1	1	3	1	4	3	4	3	1	5	3	4	1	4
2	1977	1	3	6	4	2	3	3	2	1	3	3	1	2	1	1	2	1	2	2	2	2	1	2	2	2	1	4
2	1982	1	3	80	6,5	3	4	3	3	1	4	4	1	4	1	1	3	1	1	3	1	2	1	4	2	2	3	4
1	1969	1	5	8	4	3	0	0	1	1	2	4	1	5	1	1	2	1	2	3	1	3	1	5	4	4	1	4
2	1966	1	20	3	4	4	20	15	1	1	5	5	1	5	1	1	5	1	5	1	1	5	1	1	5	5	3	5
2	1970	0	5	2	3	3	0	0	3	1	2	4	1	4	1	1	3	1	1	3	3	3	1	1	3	3	1	4
2	1973	1	10	15	3	3	3	3	4	2	4	5	1	5	1	1	4	1	4	5	1	4	1	4	4	4	2	4
2	1967	1	10	4	4	2	3	3	3	1	3	3	1	3	1	1	3	1	1	3	1	1	1	3	3	3	1	3
2	1979	0	5	8	3	3	9	7	3	1	3	3	1	3	1	1	3	1	1	3	1	3	1	3	3	3	1	1
2	1955	1	15	3	3	3	15	13	3	1	3	4	3	4	3	1	4	3	4	3	1	4	2	3	3	4	3	4
2	1971	0	0	3	3	3	0	0	1	1	2	5	3	4	3	1	4	1	3	2	3	2	2	2	2	2	1	2
2	1982	0	2	7	4	3	1	1	1	1	2	3	1	5	3	1	1	1	1	4	1	5	1	1	3	5	1	3
2	1982	0	1	4	8	1	5	0	4	1	4	2	1	1	1	1	4	1	1	3	1	1	1	1	2	2	1	1
2	1973	1	13	5	3	4	10	1	1	1	4	2	1	4	1	1	4	1	2	2	1	3	1	4	4	2	1	2
1	1967	0	15	10	4	4	12	11	5	1	5	5	1	4	2	1	4	1	2	2	1	2	1	5	5	3	1	2
1	1959	0	10	3	3	2	15	13	1	3	3	5	1	2	1	1	3	3	1	1	1	3	1	3	3	3	3	3
2	1980	1	5	25	3	2	0	0	1	3	3	2	1	2	1	1	1	1	1	2	1	1	1	2	4	4	1	3
2	1989	0	2	7	3	4	0	0	3	1	4	4	1	4	1	1	4	1	4	4	1	3	2	4	4	4	1	3

2	1963	0	5	3	4	3	4	4	1	1	2	3	1	4	3	1	2	1	3	4	1	4	1	4	4	3	1	5
2	1973	0	1	4	15	3	5	0	1	1	5	4	1	4	1	1	5	1 et	4	1	1	4	1		4	4	1	1
1	1972	0	9	12	3	4	12	8	3	1	3	5	1	5	1	1	4	1	5	5	1	5	1	5	3	4	3	4
2	1979	0	6	12	4	3	0	0	3	2	2	2	1	4	1	1	2	1	1	2	1	4	1	4	4	4	1	2
2	1962	0	10	5	3	3	5	3	1	1	1	1	1	2	1	1	2	1	3	3	1	2	1	1	3	3	1	1
2	1975	0	5	5	1	4	0	0	1	3	2	4	1	4	2	2	3	2	3	2	4	2	2	3	4	3	1	3
2	1967	0	5	7	2	1	0	0		1	2	2	1	3	1	1	2	3	2	2	3	3	1	2	5	4	1	2
2	1964	0	15	12	4	3	7	6	1	1	1	1	1	1	1	1	2	1	2	1	1	2	1	3	2	1	1	1
2	1961	0	20	3	2	2	6	4	2	3	3	4	1	4	1	1	2	3	3	3	2	2	1	4	3	3	1	2
2	1958	0	4	6	3	3	10	9	1	1	4	5	1	3	1	1	3	1	2	1	1	5	1	3				
2	1983	1	7	3	3	2	6	5	4	1	2	5	1	5	1	1	3	1	2	2	1	2	2	3	3	3	1	4
2	80	1	7	15	5	3	22	22	4	1	4	4	1	4	1	1	4	1	4	4	1	4	4	4	4	4	1	4
2	1965	0	12	8	3	3	25	22	3	1	5	5	1	3	1	1	5	1	4	3	1	3	1	3	3	3	1	3
2	1975	1	6	5	3	1	3	3	1	1	3	4	1	2	4	1	2	1	2	3	1	2	1	3	4	4	1	4
2	1974	0	5	6	3	2	2	2	2	1	1	2	1	4	2	1	1	1	2	2	1	2	1	5	5	5	1	4
2	1980	0	5	3	5	1	4	3	1	1	2	1	5	1	1	1	4	3	3	2	5	3	1	1	3	1	1	3
2	1983	0	3	6	3	2	0	0	1	1	3	4	1	2	1	1	4	3	1	1	1	2	1	4	4	4	1	3
2	1985	0	2	16	3	4	0	0	4	1	4	4	1	4	1	1	3	1	1	1	1	4	1	1	4	3	1	1
2	1969	0	22	8	3	3	4	4	1	1	4	2	1	2	1	1	4	1	4	4	1	2	1	2	4	2	1	2
2	1990	1	3	17	4	4	0	0	5	1	5	5	3	5	4	3	5	1	4	5	1	5	3	5	5	5	1	5
2	1979	0	3	12	4	2	0	0	1	1	5	5	1	5	1	1	5	1	2	2	1	3	1	4	4	4	4	3

2	1993	0	2	6	4	2	0	0	3	5	5	2	2	5	1	1	4	1	4	3	1	4	2	4	4	4	4	4
2	1972	1	15	7	2	1	10	8	4	1	4	4	1	2	1	1	4	1	4	3	1	4	1	4	4	4	1	4
2	1960	1	4	3	4	1	6	5	4	1	5	4	1	2	1	1	3	1	1	3	1	4	1	3	5	1	1	5
2	1980	0	15	8	1	5	12	12	1	1	4	5	1	3	1	1	4	1	2	1	1	5	1	2	5	5	1	4
2	1967	1	15	7	4	3	30	27	4	1	4	4	1	4	1	1	4	1	5	4	1	4	1	4	4	4	4	4
2	1948	1	9	8	3	4	9	8	1	1	3	4	1	5	1	1	5	1	4	2	1	2	1	2	4	4	1	4
2	1960	0	12	16	1	1	6	5	1	1	3	2	1	4	1	1	2	1	3	2	1	2	1	4	2	4	1	4
2	1960	0	7	10	4	4	10	6	1	1	4	3	1	1	1	1	2	1	4	1	1	2	1	4	4	4	1	4
1	1968	0	6	5,5	3	4	2	1	1	1	4	3	1	1	1	1	2	1	2	1	1	2	1	1	2	2	1	1
2	1984	0	5	9	3	3	1	1	2	1	5	5	3	4	4	1	4	1	3	2	1	2	2	2	3	2	1	2
2	1962	1	10	6	3	4	3	2	1	2	4	3	1	2	1	1	3	2	2	3	2	3	2	2	4	2	1	2
2	1965	0	32	15	3	4	15	15	4	1	1	5	1	4	1	1	2	1	5	2	1	4	1	5	4	2	1	4
2	1960	1	8	12	3	1	7	7	1	1	3	5	1	2	1	1	1	1	5	2	1	4	1	4	5	4	1	5
2	1965	0	10	25	4	5	15	15	4	1	1	2	1	2	1	1	1	1	4	1	1	3	1	3	1	1	1	1
2	1983	0	12	15	3	4	2	2	3	1	2	2	1	2	1	1	2	1	2	2	1	2	1	3	2	2	3	2
2	1983	1	7	8	4	3	6	6	3	1	3	5	1	4	1	1	2	1	4	3	1	4	1	5	2	2	1	4
2	1985	0	2	6	4	3	5	5	1	1	3	4	1	5	2	1	4	1	3	3	1	3	1	2	3	4	3	3
2	1981	1	6	10	4	4	2	2	1	1	3	1	1	4	1	1	1	1	1	2	1	1	1	4	2	3	1	3
2	1978	1	5	12	3	3	7	7	1	3	3	3	1	3	1	1	2	2	2	3	2	3	2	2	2	4	1	2
2	1984	1	4	10	3	1	4	4	3	1	5	4	1	4	1	1	4	1	2	3	1	4	1	4	4	2	1	4

1	1969	1	11	11	3	3	7	5	4	1	4	4	1	4	1	1	3	1	4	3	1	4	1	5	4	4	1	4
1	87	1	4	10	3	4	1	1	3	1	5	5	2	5	3	1	5	1	4	4	1	5	5	4	5	5	2	4
2	1977	0	5	4	4	3	3	0	4	1	4	4	3	4	1	1	4	1	3	4	1	4	3	2	2	2	1	2
2	1971	0	6	6	3	1	1	1	3	3	4	4	3	4	3	1	4	1	4	4	2	2	2	4	4	4	1	4
1	1973	0	4	4	3	3	6	5	1	1	2	2	1	4	1	1	4	1	3	4	1	3	1	4	5	4	1	4
2	1966	0	7	12,5	3	4	20	15	3	3	4	4	4	4	3	3	4	1	4	2	1	4	3	4	3	3	1	4
1	1989	0	8	4	3	3	4	4	2	1	2	5	4	3	1	1	2	2	4	4	1	3	1	2	2	2	1	4
2	1968	1	5	6	4	3	15	12	3	5	3	5	1	2	1	1	2	1	5	5	1	5	1	5	4	5	1	1
1	1962	1	13	5	3	3	18	17	3	1	3	3	1	3	1	1	3	1	3	3	1	2	1	2	3	2	1	3
2	1990	0	1	17	4	3	1	1	1	2	3	5	1	4	1	1	5	1	3	1	1	4	1	4	4	4	2	4
2	1956	0	13	8	3	3	3	2	4	1	3	5	2	5	2	1	3	1	2	5	1	5	1	5	3	4	1	4
2	1981	1	14	8	4	4	15	13	3	1	4	4	1	3	1	1	4	3	4	4	1	4	1	4	3	2	1	3
2	1974	0	5	5	3	1	4	3	1	1	5	5	1	5	1	1	5	1	1	5	1	5	1	5	5	5	1	5
2	1971	0	2	7	4	4	1	1	3	1	2	2	1	2	1	1	2	3	3	3	1	2	1	2	2	2	1	2
2	1967	1	11	6,5	3	4	6	5	1	1	4	4	1	2	1	1	2	1	5	1	1	4	1	3	4	4	1	4
1	1955	0	19	8	3	4	40	40	3	1	4	5	1	4	1	1	3	1	4	5	1	4	1	4	4	5	3	4
2	1970	1	10	17	4	3	15	14	5	1	5	4	1	5	3	1	4	1	5	3	1	5	2	5	4	5	1	4
2	1968	1	7	2	3	3	3	3	4	2	3	4	1	1	1	1	3	1	2	1	1	4	1	5	4	1	1	3
2	6	0	9	6	3	4	10	9	1	1	1	1	1	1	1	1	4	1	4	3	1	3	1	4	4	3	1	3
2	1980	1	1	5	3	3	1	1	1	1	3	1	1	1	1	1	4	1	2	2	1	2	1	3	4	4	2	2
1	1991	0	4	6	3	2	3	3	4	1	5	4	1	4	1	1	5	1	5	4	1	4	5	5	5	5	3	5
2	1967	1	1	12	4	4	0	0	1	1	1	1	1	2	1	1	1	1	1	4	1	2	1	2	2	2	1	2

2	1985	0	0	3	1	5	0	0	1	3	1	3	1	1	1	1	1	1	1	1	1	1	1	1	1	1	1	3
2	1986	0	5	10	3	5	0	0	1	3	2	1	2	2	2	2	2	2	1	2	2	3	1	1	2	3	1	1
2	1988	0	2	7	3	3	0	0	3	1	4	3	1	4	1	1	4	1	4	3	1	3	1	4	4	3	1	4
2	1960	0	5	6	3	3	0	0	2	1	4	4	1	4	3	3	3	1	3	3	1	2	3	3	2	5	1	3
2	1961	0	15	15	3	3	10	10	1	1	4	4	1	3	2	2	4	2	4	4	1	4	3	3	3	3	1	1
2	1982	0	3	6	2	2	2	2	3	1	2	3	1	2	1	1	2	1	2	2	2	3	1	3	3	3	1	2
2	1972	0	9	8	4	4	4	3	1	1	2	4	1	4	3	1	5	1	5	5	1	4	1	3	5	4	1	4
2	1982	0	4	8	2	2	3	3	3	1	5	5	1	5	1	1	5	1	2	4	1	1	1	4	5	5	1	1
2	1972	1	16	10	4	2	15	10	5	1	4	4	1	4	2	2	4	1	5	4	1	5	4	5	4	4	3	5
2	1980	0	2	7	3	3	1	1	3	1	3	5	1	4	3	1	3	1	3	4	1	3	1	3	3	3	1	4
2	1981	1	4	6	3	4	0	0	1	1	4	4	1	4	1	1	4	1	1	4	1	2	1	2	3	3	1	2
2	1972	1	4	3	3	3	3	3	1	1	1	1	1	1	1	1	1	1	1	1	1	1	1	2	1	1	2	1
2	1973	0	9	8	3	2	12	8	3	1	2	4	1	4	1	1	2	1	5	5	1	4	1	5	4	5	1	5
2	1968	1	10	2	2	2	18	15	4	2	4	5	1	4	2	1	4	1	3	2	3	3	1	4	3	3	1	2
2	1965	1	3	6	3	1	3	3	3	1	5	4	1	4	1	1	4	1	4	3	1	4	1	4	4	4	1	4
2	1969	1	4	11	3	3	5	5	4	1	4	4	1	4	1	1	4	1	4	4	1	4	1	4	4	4	2	4
2	1967	1	5	3	8	3	2	4	1	1	2	2	1	2	1	1	2	1	1	2	1	2	2	1	2	2	2	2
2	1990	0	5	12	4	4	0	0	3	1	4	5	1	3	1	1	5	1	4	4	1	4	1	5	5	3	1	3
2	1967	0	2	12	4	4	0	0	1	1	4	5	1	4	1	1	4	1	5	5	1	4	1	3	5	5	1	4
1	1974	0	2	6	2	3	1	0	2	3	2	3	1	3	1	1	4	1	2	3	3	3	1	2	3	3	2	2
2	1982	0	10	1	3	4	1	0	1	1	4	3	1	4	1	1	4	3	1	3	1	3	1	3	4	4	1	4
2	1976	1	5	6	3	2	6	5	1	1	4	2	3	4	2	1	4	1	2	2	1	3	3	2	2	2	2	2

2	1978	1	5	4	4	4	6	6	1	3	2	2	3	5	3	3	2	1	2	3	1	5	1	3	3	3	3	4
1	1966	1	7	15	5	4	1	1	1	1	1	3	1	5	1	1	2	1	3	3	1	3	1	2	2	2	1	3
2	1952	1	20	4	2	1	40	35	1	2	2	4	1	2	1	1	2	1	2	1	2	2		2	2	2	2	2
1	1972	0	3	7	3	4	2	2	3	1	2	1	1	4	1	1	4	1	1	3	1	1	1	3	4	4	1	3
2	1974	1	3	12	3	1	8	8	1	1	1	2	2	2	1	2	4	1	3	2	1	2	1	2	4	2	2	2
2	1976	0	10	15	3	4	8	8	4	1	5	5	1	4	1	1	5	1	5	3	1	5	1	5	5	4	1	5
1	1966	0	10	10	4	4	10	8	1	1	1	5	1	1	1	1	1	1	4	1	1	4	1	1	4	1	1	1
2	1973	0	3	7	3	1	0	0	3	1	2	4	1	4	1	1	4	1	1	3	1	4	4	4	4	4	3	4
1	1967	1	5	12,5	4	2	15	14	1	1	3	5	1	2	1	1	2	1	5	3	1	4	1	3	2	2	1	2
2	1990	0	4	6	4	1	1	1	1	2	5	5	1	4	1	1	4	1	3	4	1	3	1	4	4	4	1	4
2	1975	0	4	12	4	4	10	6	1	1	1	2	1	3	1	1	1	1	2	1	1	2	1	1	1	2	4	2
2	1952	1	10	15	5	5	17	16	3	1	5	5	1	3	1	1	5	1	4	1	1	4	1	4	5	1	2	4
2	1968	0	4	5	2	2	4	4	2	1	4	5	1	4	1	1	3	1	4	4	1	5	1	5	4	5	1	5
2	1964	0	10	20	4	4	0	0	5	1	4	4	1	4	1	1	4	1	4	4	1	4	4	4	4	4	2	4
2	1957	0	10	7	3	4	0	0	2	2	2	4	4	2	3	1	1	1	2	2	2	2	2	2	2	2	1	2

TPB Constructs									Coping Strategies																						
**SN2**	**ATT2**	**SN3**	**ATT3**	**SE1**	**SE2**	**INT1**	**SE3**	**INT2**	**CopActif1**	**CopRpb1**	**CopSC1**	**CopRpos1**	**CopPmagic1**	**CopCS1**	**Copdét1**	**CopBlame1**	**CopActif2**	**CopRpb2**	**CopSC2**	**CopRpos2**	**CopPmagic2**	**CopCS2**	**CopDét2**	**CopBlame2**	**CopActif3**	**CopRPb3**	**CopSC3**	**CopRpos3**	**CopPmagic3**	**CopCS3**	**CopDét3**
1	2	1	2	2	2	4	2	3	3	4	4	4	4	3	4	4	4	3	5	5	2	4	4	3	4	4	4	5	1	4	4
1	4	1	1	5	5	5	5	5	5	5	4	1	1	1	5	1	5	5	3	1	1	2	4	2	4	4	4	4	2	5	5
1	2	1	2	4	4	5	5	4	2	4	3	4	3	2	2	3	4	3	3	5	4	4	4	2	4	4	2	5	2	4	4
2	4	2	4	4	3	5	4	3	3	5	3	3	2	1	3	4	5	4	4	3	1	3	1	3	4	4	2	4	1	5	3
1	2	1	2	3	1	2	2	3	2	2	3	2	1	1	1	1	2	2	1	4	1	2	1	2	2	2	4	2	1	3	2
1	5	3	2	4	4	5	5	4	1	4	4	4	5	5	5	5	5	5	5	5	5	5	5	3	5	5	4	5	1	4	5
1	4	1	1	4	4	5	4	3	1	4	5	3	1	1	2	1	3	1	4	5	1	4	3	3	5	4	3	4	1	4	4
1	3	1	2	4	3	5	5	3	4	4	4	4	2	3	4	4	4	4	4	4	3	4	4	3	4	4	4	4	3	4	4
2	2	1	2	4	3	4	4	3	4	4	4	3	4	4	3	4	3	3	3	3	1	2	2	3	3	3	2	3	2	2	2
1	1	3	3	5	5	5	5	4	4	4	5	4	5	3	4	4	4	4	5	4	3	3	3	4	4	4	4	4	4	4	4
4	4	2	1	4	4	4	4	4	4	3	2	3	1	1	2	2	3	2	1	3	2	3	2	3	1	2	3	4	1	5	5
1	2	1	1	4	2	5	4	3	2	1	4	4	4	1	4	4	1	4	3	4	4	4	1	2	3	2	4	2	1	5	5
1	4	1	2	4	3	5	5	3	5	5	5	4	5	1	3	4	4	4	4	3	3	4	4	1	4	4	3	5	1	4	4
1	4	1	4	4	5	4	5	4	3	4	4	3	2	1	4	1	2	1	4	4	4	4	5	1	4	4	4	4	1	4	4
1	5	1	1	5	3	5	5	3	3	3	4	3	3	1	3	3	3	3	3	5	3	3	3	5	3	3	3	3	4	3	3
1	2	1	1	2	3	2	2	3	4	4	4	4	4	1	3	1	2	4	4	4	3	3	1	3	3	3	3	3	3	3	3
1	4	1	1	5	5	5	5	4	5	4	5	4	4	1	2	5	5	3	2	4	1	2	1	3	3	3	3	5	1	3	3
1	4	1	3	4	3	5	4	4	4	4	4	2	4	1	4	1	1	2	4	1	1	4	2	2	3	2	3	2	1	4	4

1	2	1	1	5	5	5	5	4	5	3	2	4	2	1	2	2	3	4	1	1	1	5	3	2	1	4	3	4	1	5	5
1	5	1	5	5	3	5	5	3	5	5	5	5	5	5	5	3	5	1	5	5	1	5	1	5	5	4	4	5	1	5	4
3	2	3	2	2	5	5	5	4	4	4	5	4	3	3	4	4	4	4	5	4	3	4	3	3	5	4	4	4	3	4	4
1	4	1	3	5	5	5	5	4	4	4	5	4	5	4	3	5	5	3	4	4	1	5	3	1	1	4	5	5	4	5	5
3	4	1	1	5	4	5	5	3	5	5	4	4	4	4	4	2	4	2	2	4	4	4	3	4	4	4	4	5	1	4	4
4	5	3	1	4	5	5	5	4	4	4	2	4	4	2	2	2	2	2	4	2	1	5	3	3	4	4	3	4	4	4	4
1	4	1	2	4	4	4	4	3	4	4	4	4	4	3	4	4	4	4	4	4	3	4	3	1	4	4	4	4	3	4	4
1	2	1	3	3	2	4	4	3	4	4	5	3	4	4	4	4	4	4	4	4	3	3	1	2	4	5	4	4	2	4	4
3	4	1	4	5	4	5	5	4	5	5	5	5	5	3	4	3	4	4	5	5	4	5	4	4	5	4	5	5	5	5	5
1	2	1	2	4	4	4	4	4	3	3	5	4	4	1	2	2	2	2	4	4	2	4	2	2	2	2	3	3	4	4	3
1	4	1	3	5	4	5	5	5	3	4	4	3	3	2	3	5	3	4	2	1	1	4	5	2	3	4	1	4	3	4	4
2	4	1	3	4	4	5	4	4	4	4	5	4	3	3	4	5	5	4	5	3	3	4	3	5	4	3	5	4	2	4	4
3	3	3	1	2	3	2	2	4	4	1	2	3	2	1	2	2	2	2	2	2	1	4	1	1	4	2	1	4	2	4	4
1	4	1	2	4	5	5	5	4	4	4	4	4	1	2	3	3	4	4	1	4	1	4	3	3	4	4	2	4	3	4	4
1	4	1	3	5	5	5	5	5	5	5	5	3	4	4	5	5	5	3	5	4	2	5	4	2	5	4	5	4	5	4	4
1	3	1	1	5	3	5	5	4	3	4	4	4	4	2	4	1	4	2	3	4	1	4	3	1	5	3	3	3	3	3	3
1	4	1	1	4	4	5	4	3	4	4	2	4	2	1	3	1	2	3	4	3	1	4	1	1	4	4	2	2	4	4	4
1	3	4	2	3	2	2	2	2	4	3	4	3	3	4	4	4	4	4	4	4	3	4	2	2	3	2	2	4	4	4	2
1	1	1	5	3	5	3	3	3	5	5	5	5	5	5	5	5	5	5	1	5	5	5	5	5	5	5	5	5	5	5	5

1	4	1	3	4	4	5	1	3	5	5	5	5	5	3	2	5	5	4	5	5	4	4	3	5	5	5	5	4	4	4	4
4	5	3	4	5	5	5	5	5	4	4	5	4	5	1	5	5	4	4	5	4	1	4	4	1	4	4	5	5	3	5	4
3	5	2	5	5	5	5	5	4	4	5	4	4	2	2	3	4	4	2	4	5	1	4	2	4	4	3	4	3	2	4	4
3	3	1	1	5	5	5	5	5	3	3	4	3	3	4	4	5	3	2	3	3	5	5	4	1	1	2	1	5	4	1	3
3	4	3	1	4	3	5	4	4	4	3	3	4	4	1	4	2	3							2	3	4	4	2	4	4	4
1	4	1	1	4	4	5	5	5	4	4	4	3	5	1	3	1	1	3	5	3	1	4	3	1	3	3	3	2	1	4	4
1	2	1	5	3	5	4	3	3	5	4	5	2	1	1	2	4	3	4	5	5	1	4	4	4	4	4	3	2	1	4	4
2	3	2	1	4	4	4	3	3	3	4	4	4	2	1	4	3	4	4	4	4	3	4	1	3	4	4	4	4	4	4	4
1	5	1	3	4	5	5	4	3	5	5	5	5	5	2	5	1	5	5	5	5	4	5	5	4	3	5	2	5	2	5	2
1	4	1	3	5	5	5	5	4	5	4	4	4	5	2	1	4	3	4	5	3	1	4	1	1	4	3	3	5	1	4	3
2	5	2	3	5	4	5	5	4	4	4	4	5	4	3	4	4	4	4	5	4	3	4	3	1	4	4	4	4	4	4	4
1	2	1	4	5	4	5	5	4	4	4	4	4	3	3	4	4	4	3	4	4	3	2	2	3	2	2	3	4	1	4	4
1	4	1	3	5	5	5	5	4	1	4	5	5	3	2	5	4	5	5	5	2	1	2	2	4	5	4	4	5	2	4	4
1	4	1	1	5	5	5	5	4	3	2	3	2	3	1	1	4	4	2	4	2	3	4	3	2	1	4	1	4	3	4	3
3	4	3	4	4	3	4	4	4	4	4	2	4	2	2	3	2	2	4	4	4	1	4	2	2	4	4	2	4	5	4	4
5	5	5	1	5	5	5	5	4	3	1	5	5	1	1	5	1	5	2	5	1	1	2	1	4	1	1	1	5	1	5	5
1	4	1	2	4	4	5	5	4	4	2	4	3	3	1	1	1	3	5	5	3	1	5	1	2	4	4	4	3	1	5	1
1	2	1	2	2	2	5	5	4	4	4	4	4	4	2	2	4	4	2	4	5	1	4	2	1	4	3	3	4	1	5	4
4	4	4	2	5	4	5	5	4	2	2	4	2	2	3	2	3	3	4	4	4	2	3	3	3	4	4	4	3	2	4	4

1	2	1	1	2	2	5	5	3	5	5	4	4	4	1	5	5	3	5	3	5	1	5	5	3	4	4	4	4	4	5	5
1	4	1	3	4	3	5	5	4	3	3	3	4	4	3	3	1	4	4	5	4	1	4	2	2	3	3	2	4	3	4	4
1	4	1	1	5	3	5	5	3	5	4	4	4	1	1	4	4	4	4	4	4	2	4	1	3	1	4	2	4	1	4	4
3	2	1	3	2	4	3	3	2	3	2	2	5	2	4	4	2	3	2	4	3	3	2	3	4	4	4	4	4	4	3	3
2	4	2	1	4	4	5	4	4	4	4	4	3	3	1	2	1	2	5	4	5	2	4	2	2	2	3	4	3	1	4	4
1	2	1	3	4	4	5	4	3	5	5	4	4	4	2	4	5	4	3	4	4	1	5	4	5	4	4	4	4	2	4	4
2	4	2	3	5	4	5	5	4	3	4	4	4	1	2	3	2	3	2	2	4	1	4	1	2	3	3	5	4	1	3	3
1	4	2	3	4	5	5	4	3	4	1	3	3	4	1	4	3	4	4	4	5	4	4	1	5	4	4	4	4	4	4	5
1	3	1	2	4	4	5	4	4	4	4	4	4	4	4	4	2	5	5	5	2	1	4	3	1	4	4	4	4	2	4	5
1	3	1	1	4	3	5	5	4	4	5	3	3	3	1	4	1	3	3	3	4	1	4	3	3	4	3	5	2	2	4	2
2	4	2	3	5	3	5	5	4	5	4	5	5	5	4	5	5	5	3	5	5	4	5	4	1	5	4	5	5	2	5	5
3	3	2	2	3	3	3	3	3	2	2	3	3	2	2	3	2	2	2	4	3	2	3	2	4	3	2	4	2	2	4	2
2	2	2	5	5	5	5	5	5	3	2	5	2	2	2	2	5	5	2	5	5	2	5	2	5	3	3	5	3	1	5	5
1	3	1	3	4	5	5	5	4	4	4	5	5	4	3	4	2	3	4	5	4	4	5	4	2	4	4	4	2	4	5	4
1	2	1	5	1	1	5	2	2	2	5	2	1	2	4	4	5	4	3	2	4	1	2	1	1	2	1	5	2	1	4	2
1	5	1	4	5	4	5	4	4	3	3	5	5	4	1	5	5	3	4	5	5	1	5	1	2	5	4	5	5	4	5	5
1	2	2	1	3	4	4	3	3	3	3	2	2	4	3	4	4	4	4	4	4	4	4	4	5	4	4	4	4	4	4	4
1	4	1	1	4	2	4	4	4	3	4	4	3	3	2	3	3	3	3	1	3	1	2	2	3	3	2	2	3	3	4	3
2	4	2	3	3	2	2	2	3	2	2	4	4	4	4	4	4	4	4	4	4	3	3	3	2	4	3	3	3	3	4	3
3	3	4	4	4	4	4	4	3	4	4	4	4	4	4	4	4	4	4	4	4	4	4	4	4	4	4	4	4	4	4	4

1	3	1	4	2	4	1	3	3	4	4	1	2	1	2	2	4	4	4	1	5	1	4	4	2	3	3	1	2	1	4	4
1	4	1	1	5	3	5	5	4	5	1	3	4	1	1	2	1	2	2	1	2	2	5	1	2	2	2	4	2	1	4	3
2	5	1	1	5	4	5	5	3	2	1	4	1	4	1	4	1	2	1	5	4	3	4	2	3	1	1	4	1	2	5	3
1	4	1	4	5	4	5	5	4	4	3	5	4	5	4	4	4	4	4	4	5	2	3	3	3	4	4	5	5	3	4	2
1	1	1	1	4	4	5	5	3	2	4	3	2	1	1	2	4	2	4	5	5	4	4	2	4	3	4	4	4	1	4	4
1	3	1	4	3	4	3	3	2	4	4	5	4	2	1	1	3	1	2	2	5	4	4	3	1	3	3	3	4	2	2	2
1	2	1	3	4	4	4	4	4	3	3	4	4	2	3	3	1	3	4	3	5	2	4	1	4	3	3	2	2	3	4	4
1	4	1	5	1	3	3	3	1	4	5	5	2	1	3	4	5	5	5	1	5	1	5	4	5	4	4	5	5	2	4	4
1	5	1	3	5	5	5	5	4	4	4	5	5	1	4	4	1	3	4	4	3	1	4	1	1	4	4	4	4	1	2	2
1	3	1	1	5	5	5	5	4	3	2	4	3	1	3	4	2	1	2	1	1	1	3	2	1	3	1	2	1	1	3	3
1	4	1	1	5	4	5	5	4	2	5	4	1	4	2	2	1	4	1	4	4	1	1	1	1	4	2	3	2	2	3	2
1	3	2	2	4	4	4	4	3	4	4	5	1	2	1	2	4	4	4	4	2	3	4	3	4	4	4	4	4	1	4	4
2	4	2	4	5	5	5	5	4	4	2	5	5	5	3	2	3	2	3	5	5	2	5	2	1	5	4	3	4	3	2	2
2	4	1	1	5	4	5	5	4	3	4	3	2	3	2	2	4	2	4	5	4	4	4	2	3	4	4	4	4	4	4	4
1	3	1	3	4	5	5	5	4	3	5	5	4	3	4	4	4	5	4	5	5	3	3	4	4	4	4	4	4	4	4	3
1	3	1	3	5	4	5	5	4	5	5	5	5	3	3	3	3	4	4	5	5	1	4	1	2	2	4	4	4	2	5	4
1	2	1	1	2	3	4	3	3	4	3	3	3	1	2	4	3	4	4	4	4	1	4	1	4	4	4	3	4	3	4	2
1	3	1	5	3	3	5	4	3	5	5	3	4	1	1	1	4	4	5	1	3	3	4	3	3	3	3	3	4	4	2	4
1	3	3	5	4	1	5	5	2	4	4	5	5	5	3	5	5	5	5	1	5	1	2	4	5	4	5	3	5	3	3	4
4	4	1	4	5	5	5	5	4	5	5	4	4	4	3	4	4	5	4	5	4	3	4	4	2	3	4	4	4	3	4	4
1	5	2	5	1	5	5	4	4	2	5	2	3	1	1	4	1	5	1	2	5	1	4	2	3	5	4	4	5	1	5	5
1	2	1	1	5	1	5	5	5	2	4	4	2	4	1	2	1	2	4	4	4	1	4	1	1	2	2	1	2	2	2	4

1	2	1	3	2	2	4	4	3	5	5	5	5	5	1	4	4	2	4	5	4	2	4	2	2	4	4	4	5	2	5	5
4	4	1	4	4	4	4	4	3	4	4	4	4	1	1	1	1	4	4	4	4	1	4	1	1	4	4	4	2	1	4	4
1	4	2	3	5	5	5	5	4	5	4	4	4	5	3	4	5	4	5	4	3	4	4	4	2	4	5	4	5	1	4	5
2	2	3	2	5	4	5	5	5	4	2	4	4	4	2	2	2	1	1	4	2	3	4	1	2	2	2	4	4	1	2	2
1	4	1	4	5	4	5	5	4	3	5	5	4	4	3	4	2	2	4	3	4	3	4	4	4	4	4	4	4	4	4	4
1	5	1	3	5	5	5	5	5	4	3	4	2	4	2	3	4	4	4	5	4	1	4	3	1	3	4	4	5	1	4	4
1	4	1	3	5	5	5	4	3	4	5	5	4	1	2	2	4	4	4	4	4	1	5	1	1	3	4	4	4	1	4	3
1	4	1	3	4	2	5	4	3	5	4	4	2	2	3	1	4	3	3	4	4	1	5	2	2	3	3	4	3	1	4	4
1	5	1	2	4	4	5	5	4	5	4	5	3	4	3	3	2	2	4	5	4	2	5	2	2	3	3	4	4	4	5	5
3	5	3	3	5	5	5	5	5	5	5	5	5	5	1	5	1	1	5	5	5	1	5	1	1	5	5	5	5	5	5	5
1	4	1	1	4	2	5	5	3	2	4	3	2	3	2	4	3	3	3	2	5	2	4	2	3	3	3	4	3	5	3	2
1	4	1	4	4	3	5	5	4	4	3	4	4	3	3	3	3	3	4	4	4	3	4	3	3	4	4	4	4	2	4	4
1	3	1	3	4	3	4	4	4	3	2	1	3	2	1	1	2	2	2	3	4	1	4	2	2	2	3	3	2	1	3	3
1	3	1	3	3	4	4	4	3	3	4	3	3	2	1	3	3	4	4	1	3	1	3	1	3	3	4	3	2	1	4	3
2	4	3	2	3	4	5	4	3	4	3	4	3	4	2	4	3	3	4	4	4	3	4	3	4	4	3	4	4	2	4	4
3	2	1	3	4	4	5	5	4	2	5	4	4	4	3	3	5	5	4	5	5	2	3	3	2	4	4	4	4	3	4	4
1	5	1	3	4	4	4	4	4	4	4	3	1	5	3	3	4	5	3	5	5	2	5	3	2	5	4	4	2	3	5	4
1	1	1	1	2	4	2	3	3	2	4	2	2	1	2	1	4	4	1	1	4	1	2	2	2	4	4	2	2	2	2	2
1	2	1	2	3	3	4	4	3	4	4	4	4	3	3	4	4	2	4	5	4	3	5	4	4	4	4	3	3	1	4	4
1	2	1	2	4	4	5	5	5	5	4	4	4	4	2	2	4	4	4	4	4	2	4	2	3	4	4	4	4	2	4	4
3	2	2	3	5	5	5	5	5	5	5	5	5	5	5	5	5	5	5	5	5	5	5	4	4	5	5	5	4	4	5	5
1	1	1	4	3	4	4	4	3	4	4	4	4	3	2	2	4	4	4	5	5	1	4	3	4	4	4	4	5	1	2	3
1	3	1	3	4	1	5	4	4	4	5	2	3	4	2	4	4	3	4	4	4	4	3	3	4	4	4	2	2	1	3	4

1	5	1	1	5	5	5	5	5	4	4	5	4	5	3	1	3	3	4	4	5	4	4	4	3	3	4	4	4	5	4	5
1	1	1	1	3	4	2	2	3	5	5	5	3	2	1	4	2	3	5	2	3	1	4	2		2	3	2	3	1	4	3
4	4	4	4	5	3	5	5	3	5	4	5	4	5	1	5	4	4	5	1	1	1	3	1	1	4	5	3	4	1	4	3
1	2	1	2	2	1	5	3	3	1	3	4	3	4	1	2	4	2	2	5	1	5	5	2								
1	3	1	2	2	2	2	2	4	4	3	3	3	2	2	2	4	4	4	1	4	2	3	4	3	4	4	4	4	3	4	4
1	4	1	3	2	4	1	1	1	1	4	3	4	2	3	3	3	4	4	3	4	2	4	2	3	4	3	4	4	3	4	3
3	2	3	2	3	4	2	2	3	4	4	4	4	4	3	3	4	4	4	4	4	1	4	4	3	4	4	4	4	3	4	4
1	2	1	1	2	4	4	4	4	3	3	2	3	1	1	1	3	3	3	4	4	1	4	1	1	4	4	4	4	3	4	4
1	2	1	1	4	4	4	4	3	4	4	4	4	4	1	1	4	3	4	4	4	3	4	4	4	4	4	4	4	4	4	4
	4	1	1	5	2	5	5	3	1	3	2	4	2	4	3	2	3	4	1	4	3	4	4	1	4	3	1	4	1	4	4
1	4	1	3	5	4	5	5	5	5	4	5	4	5	4	4	4	4	4	5	5	3	5	1	2	5	5	3	5	2	5	4
1	4	1	4	5	5	5	5	5	5	3	4	1	1	1	1	1	1	1	1	1	1	4	1	1	1	1	1	1	1	4	4
1	3	1	1	5	5	5	5	5	3	3	3	5	1	2	3	5	5	5	5	4	1	4	2	2	2	2	2	5	2	4	3
2	4	3	3	5	4	5	4	3	4	4	5	3	4	5	4	3	4	5	4	5	5	2	4	4	4	4	4	5	3	4	4
1	5	1	4	4	5	5	5	4	2	4	4	4	3	2	1	4	3	5	4	4	1	5	2	1	3	4	2	4	2	3	3
1	4	1	5	4	3	4	4	3	5	5	5	3	4	1	4	5	3	3	5	5	1	4	5	5	5	5	5	3	1	5	5
1	3	1	4	5	5	5	5	4	4	4	1	4	4	1	4	4	4	4	1	4	1	4	1	1	4	4	1	4	4	4	4
1	1	1	4	3	4	4	3	3	5	2	4	3	4	1	4	4	4	4	4	4	1	4	4	4	4	4	4	4	4	4	4
1	4	1	3	4	4	5	4	3	5	4	5	3	1	1	4	5	5	2	4	5	2	5	3	1	4	1	4	2	1	5	5
1	5	1	1	4	5	5	5	4	5	5	5	5	3	1	5	5	5	5	5	5	2	5	1	5	5	5	5	5	1	5	5
2	3	2	1	5	5	5	5	4	4	4	4	4	4	3	2	2	5	4	4	5	3	4	2	3	1	4	4	4	3	4	3

3	4	2	4	4	4	5	4	3	1	2	5	5	4	1	1	1	3	4	2	5	1	2	4	4	1	2	4	4	4	4	3
1	4	1	3	5	4	5	5	5	5	5	4	4	5	3	3	1	4	4	5	4	1	4	1	1	4	4	4	3	3	4	4
1	5	1	1	5	5	5	5	4	5	1	3	5	1	5	5	5	3	5	1	5	1	3	1	1	5	3	1	5	1	5	5
1	4	1	3	5	5	5	5	5	5	4	4	4	4	1	2	4	5	4	3	3	1	5	5	1	1	4	4	4	1	5	5
2	4	1	1	5	5	5	5	4	5	5	4	5	5	2	5	5	5	4	5	2	1	5	3	1	3	5	5	4	1	5	5
1	5	1	2	5	4	5	5	4	4	5	4	3	5	4	1	4	4	4	3	4	1	5	1	1	5	4	2	4	1	4	4
1	5	1	1	3	5	4	3	2	3	3	5	3	1	2	4	1	2	3	1	4	2	3	4	1	2	2	4	2	1	3	3
1	4	1	1	4	4	5	4	4	4	4	4	4	4	1	2	4	2	5	1	4	1	5	1	1	4	4	2	4	1	5	4
1	2	1	2	2	2	2	2	3	5	5	5	3	4	1	2	5	5	5	5	5	3	3	1	2	5	5	5	5	3	4	3
1	2	1	5	3	3	5	3	3	3	5	3	3	3	4	4	5	5	5	5	5	5	5	5	5	5	5	5	5	5	3	3
1	2	1	3	4	2	5	5	4	3	3	4	3	2	3	3	3	4	3	4	4	2	3	1	4	3	3	4	4	1	4	2
1	4	1	1	4	4	5	4	4	3	3	4	2	4	1	3	2	2	4	3	3	1	4	2	4	4	3	4	3	1	4	1
1	5	1	4	5	5	5	5	5	3	5	5	5	5	1	1	5	5	3	1	5	1	5	1	2	5	2	4	5	4	3	3
1	3	1	1	5	5	5	5	5	3	1	4	4	1	1	4	4	1	1	3	2	1	4	4	1	2	1	3	1	3	4	4
3	2	3	3	4	4	4	4	4	3	2	3	2	1	1	3	3	5	4	4	4	2	4	3	4	4	4	1	2	1	4	4
1	5	1	2	5	4	5	5	5	5	4	4	4	5	1	3	4	4	4	5	5	3	5	3	1	5	4	3	4	1	5	4
3	3	3	4	5	4	5	4	3	5	4	4	2	4	2	3	4	4	4	5	4	3	4	3	4	4	4	4	4	3	3	3
1	4	1	4	3	3	4	4	3	3	4	4	3	2	1	4	4	3	2	4	4	1	4	1	3	4	3	4	1	1	4	4
1	2	1	3	3	1	3	3	3	3	4	4	3	3	2	2	2	3	4	4	3	2	4	3	4	3	3	3	1	1	3	2
1	4	1	3	4	4	5	5	4	3	4	4	4	3	3	4	3	4	4	3	3	1	3	3	2	3	3	3	4	2	4	3

1	5	1	3	4	3	5	4	4	3	4	3	5	3	4	3	1	4	4	2	2	4	5	3	3	4	4	4	4	3	5	5
3	5	3	2	5	5	5	5	4	3	4	5	4	5	3	2	1	3	5	5	4	3	5	2	1	5	4	4	5	3	5	3
3	2	3	2	3	2	2	2	3	3	3	2	4	3	5	4	4	4	4	4	4	3	4	4	4	4	4	4	4	2	4	4
1	4	1	1	4	4	5	4	4	4	4	5	3	5	4	3	4	4	4	4	4	3	3	3	4	4	4	4	4	4	4	4
2	4	3	2	3	4	5	2	3	3	4	2	4	4	4	3	4	4	3	1	5	3	4	4	2	3	3	4	3	2	4	4
3	4	3	3	4	4	4	4	4	2	5	3	3	3	1	4	4	4	4	3	4	3	4	4	3	4	4	2	2	2	4	4
1	4	1	1	2	2	5	4	3	4	5	5	5	4	1	2	5	4	3	4	4	1	5	1	2	2	2	2	1	5	4	4
1	5	1	5	5	5	5	5	5	5	5	5	5	5	1	5	1	4	2	4	4	1	5	4	4	4	5	5	5	3	4	5
1	3	1	3	5	5	5	5	4	4	4	4	4	4	1	4	1	4	4	4	4	1	2	2	1	2	4	4	1	2	2	1
2	4	2	4	5	3	5	4	4	5	5	5	4	5	3	3	3	3	5	5	5	3	4	1	1	5	4	1	2	5	4	4
3	4	1	3	4	5	5	3	3	5	5	5	4	1	3	4	5	3	4	5	4	1	4	1	3	3	3	4	5	1	5	5
1	3	1	3	4	4	5	5	4	5	5	3	3	3	3	4	1	5	4	4	4	3	5	3	2	4	4	3	5	1	5	4
1	5	1	5	4	4	5	4	3	4	4	5	4	1	1	5	5	5	4	1	5	4	5	4	5	5	5	5	5	1	5	4
1	2	1	1	2	2	4	4	4	4	4	3	3	1	1	3	2	4	5	4	4	1	4	2	1	1	4	2	1	2	4	4
1	4	1	1	5	3	5	5	4	5	5	5	5	4	1	4	4	4	4	3	4	1	5	4	3	3	4	4	4	4	5	4
4	4	3	3	5	5	1	5		4	4	3	4	4	4	3	4	4	4	1	4	4	5	1	1	4	4	4	4	1	4	3
1	4	1	1	5	5	5	5	5	5	5	4	4	4	3	4	3	4	5	5	5	2	3	3	4	4	5	4	4	4	5	4
1	5	1	1	5	4	5	5	5	4	3	4	5	5	1	1	1	1	4	4	4	1	5	1	2	4	2	5	3	1	4	3
1	4	3	1	4	1	5	5	3	2	3	3	3	4	4	4	4	4	4	4	4	4	4	4	2	3	3	1	1	1	3	3
2	2	3	1	5	5	5	5	4	5	5	5	5	5	1	1	5	5	4	4	4	3	5	2	3	3	5	4	4	3	5	5
4	5	3	1	5	5	5	5	4	5	3	4	3	5	3	4	5	5	4	5	5	3	4	5	2	5	3	5	5	3	4	4
1	2	1	1	4	2	4	4	4	4	4	2	1	1	1	3	1	1	4	2	2	1	4	3	1	1	4	3	4	1	4	4

1	5	1	5	5	3	5	4	3	2	5	2	5	3	1	3	3	4	4	4	4	1	2	2	4	3	3	4	2	3	5	3
1	2	1	2	4	4	3	4	3	4	4	4	2	2	2	3	3	3	3	4	4	1	2	1	3	4	2	2	2	2	2	3
1	4	1	3	4	4	5	4	3	3	3	2	3	4	2	2	3	4	4	3	3	1	4	2	3	4	3	3	4	2	4	3
1	3	1	3	4	3	2	4	4	3	3	1	5	1	2	3	5	5	4	1	5	3	4	3	3	5	3	2	5	3	5	5
1	2	2		4	4	5	4	3	2	3	3	4	2	3	1	3	3	4	4	2	3	3	2	2	2	3	2	2	3	4	1
2	4	1	2	4	5	5	3	3	2	3	4	2	3	1	2	2	2	2	4	2	1	3	2	4	4	5	5	3	2	3	4
1	3	1	3	4	5	5	5	5	4	3	4	3	3	2	3	2	4	4	4	4	4	2	5	4	4	5	4	5	3	5	4
1	5	1	2	5	5	5	5	4	5	5	4	5	4	3	2	1	5	3	5	4	1	5	1	1	5	4	3	5	1	3	4
3	5	3	1	5	4	5	5	4	4	5	3	4	4	4	3	5	4	4	4	5	1	4	1	1	5	5	4	5	5	4	4
1	4	1	3	4	4	5	4	4	3	3	4	3	4	2	3	5	4	3	5	3	4	4	3	5	4	3	2	4	4	4	3
1	3	1	2	4	3	4	3	3	3	3	4	2	4	1	1	4	4	4	4	4	2	4	4	3	4	4	4	5	4	4	4
1	4	1	1	5	4	5	5	5	1	4	5	5	1	1	2	3	4	4	5	5	2	4	3	5	4	1	4	1	1	5	5
2	5	2	3	4	2	5	5	4	2	4	5	4	5	2	2	5	4	4	4	5	3	4	3	4	4	4	3	5	3	4	3
1	4	1	2	4	3	5	4	4	3	4	5	3	1	3	4	1	4	5	3	5	2	5	4	5	4	4	4	4	3	4	4
1	4	1	1	5	5	5	5	4	5	4	4	4	4	3	3	2	4	4	4	5	5	5	3	2	2	4	4	4	5	5	5
2	4	2	1	5	4	4	4	4	3	4	4	4	4	2	4	4	3	2	3	4	4	4	4	2	4	4	3	3	1	4	4
2	2	2	3	3	1	2	3	3	2	4	4	2	2	2	2	2	2	2	3	4	4	2	2	2	2	2	2	2	3	2	2
1	4	1	3	4	4	5	5	4	5	4	3	3	2	2	4	5	4	4	2	4	1	3	3	4	4	4	4	5	1	3	2
1	4	1	1	5	5	5	4	4	5	5	3	4	4	1	4	4	4	4	4	4	1	4	1	2	4	4	4	4	1	4	4
1	3	1	1	3	5	4	3	3	3	4	5	3	3	2	4	5	5	4	5	5	4	4	4	5	4	4	3	5	5	4	4
1	4	1	4	5	5	5	5	4	3	4	4	4	4	4	1	5	5	5	4	5	4	5	5	3	1	4	3	5	3	4	5
2	2	1	2	4	4	5	5	4	4	4	4	4	4	3	3	4	5	4	5	5	2	5	3	2	4	4	4	4	2	3	3

3	4	4	4	4	2	5	5	4	3	3	3	3	5	5	5	4	3	3	3	3	3	3	4	3	3	3	3	3	3	3	3
1	2	1	2	5	2	5	5	4	4	1	4	4	1	3	3	1	4	5	4	4	3	4	2	2	5	5	5	5	3	5	5
2	2	2	2	2	2	2	2	3	2	3	3	4	2	2	2	2	1	1	2	2	1	2	2	1	2	2	2	1	1	2	2
1	4	1	1	4	5	5	4	3	3	5	4	4	4	3	3	3	3	3	4	4	1	5	3	4	2	3	3	2	1	3	3
2	2	2	1	5	3	5	5	4	5	4	4	2	1	1	1	1	1	4	1	2	2	1	1	1	3	2	3	3	1	4	2
3	5	1	1	5	4	5	5	4	3	4	4	3	4	1	3	3	2	2	4	2	1	4	3	1	4	3	2	4	1	5	5
1	1	1	1	5	4	4	5	4	5	5	5	4	3	1	5	5	5	4	4	5	4	5	3	2	5	5	5	4	3	5	5
3	3	3	2	4	5	5	5	3	5	4	5	4	4	2	2	2	4	4	5	5	3	4	3	2	4	4	4	4	4	5	5
3	2	1	2	5	2	5	4	4	1	4	4	4	4	1	4	4	4	4	4	5	4	4	4	1	4	5	5	4	3	4	5
1	4	1	3	5	3	5	5	4	1	4	3	4	4	3	4	2	3	4	3	3	1	3	2	3	4	4	4	4	2	3	3
1	2	2	1	5	5	5	5	5	5	5	4	4	1	4	3	4	4	5	5	3	4	4	4	2	3	4	3	4	4	4	4
1	3	1	3	5	5	5	5	5	5	1	2	1	1	1	1	1	1	1	3	3	3	3	1	4	1	1	3	1	1	3	4
1	4	1	2	3	4	5	3	3	2	2	4	3	5	2	3	2	4	4	3	5	4	5	4	4	4	4	4	4	3	4	4
1	4	1	1	5	5	5	5	5	5	5	5	5	5	1	5	5	5	5	5	5	5	5	5	3	5	5	5	5	5	5	5
3	2	1	2	2	2	2	2	3	4	4	2	4	2	2	2	2	2	4	2	4	2	4	2	4	2	2	2	2	4	4	4

	Basic psychological needs (needs satisfaction)															Achievement goals																		
CopBlame3	NS1	NS2	NS3	NS4	NS5	NS6	NS7	NS8	NS9	NS10	NS11	NS12	NS13	NS14	NS15	MG1	PG1	MG2	PG2	MG3	PG3	MG4	PG4	MG5	PG5	MG6	PG6	MG7	PG7	MG8	PG8	MG9	PG9	MG10
1	5	5	3	5	5	4	5	4	4	5	4	1	5	4	1	4	3	3	3	4	3	4	3	4	3	4	3	4	3	4	3	4	2	4
1	5	4	1	5	5	4	5	5	5	5	5	1	5	5	1	5	1	4	1	5	1	5	1	5	1	4	1	4	1	4	1	4	1	4
2	5	5	3	5	5	4	4	4	4	5	4	1	5	5	4	5	2	4	1	5	1	5	1	5	1	4	1	5	2	5	1	5	2	4
3	5	5	4	5	4	3	4	4	4	4	2	3	4	4	3	4	1	4	1	5	1	5	1	5	1	4	1	5	1	4	1	4	1	4
1	2	2	3	4	4	2	4	4	3	4	4	3	4	4	3	2	1	2	1	2	1	4	1	4	1	2	1	2	1	2	1	4	1	3
4	5	2	3	5	3	3	5	4	4	4	4	3	3	4	2	5	3	3	3	4	1	5	1	5	1	3	1	5	1	5	1	5	1	5
1	5	5	2	5	4	3	5	4	3	5	4	1	5	4	1	3	1	3	1	5	1	5	1	5	1	3	1	5	1	5	1	5	1	4
2	4	5	3	3	4	4	4	4	4	5	4	1	4	4	2	4	1	4	1	5	1	4	1	5	1	2	1	4	1	3	1	4	1	4
2	4	4	2	4	4	3	4	3	3	4	3	1	4	4	3	4	5	3	3	3	3	3	3	3	4	4	3	4	1	4	2	4	1	4
3	4	4	4	4	4	4	4	4	4	4	4	3	1	3	4	4	4	4	4	4	4	4	4	4	4	4	4	4	3	4	4	4	4	4
4	4	4	3	4	4	3	4	4	3	4	4	1	4	4	3	4	3	4	3	4	3	4	4	4	3	3	3	4	4	4	3	4	3	4
1	5	5	3	3	5	3	5	4	3	4	4	3	4	4	2	5	1	3	3	2	1	2	1	2	1	1	1	3	1	2	1	5	1	2
2	5	4	4	4	4	4	4	3	4	4	2	3	4	4	4	4	1	4	4	5	1	4	1	5	1	2	1	4	1	4	2	5	2	4
1	5	4	1	4	5	4	5	4	4	4	4	4	4	4	1	4	4	4	4	2	2	4	4	4	4	4	4	3	3	2	2	2	2	2
5	5	4	3	3	3	3	5	2	1	5	3	3	5	5	3	5	2	3	2	3	3	5	3	5	1	3	3	3	3	3	3	5	1	1
1	5	4	3	5	5	4	4	4	3	4	4	3	5	5	1	4	1	3	1	3	1	1	1	4	1	3	1	2	1	2	1	3	1	2
1	4	5	3	4	5	4	5	4	3	5	4	3	5	4	3	5	3	4	4	5	1	5	1	5	1	4	3	5	1	5	1	5	1	4
1	5	5	2	4	4	4	5	5	4	5	5	1	4	5	4	4	1	2	1	4	1	5	1	5	1	1	1	2	1	5	1	5	1	4

2	5	4	3	5	2	2	5	4	4	5	4		5	4	3	4	1	1	1	2	1	4	1	4	1	1	1	1	1	3	1	2	1	2
5	5	3	1	4	3	5	1	5	1	4	5	4	2	2	3	5	1	1	1	2	1	5	1	1	1	3	1	1	1	1	1	5	1	5
3	5	4	3	5	5	4	4	4	2	5	4	3	4	4	2	5	3	4	1	5	1	4	1	5	3	4	3	4	1	2	2	4	3	4
1	5	5	1	4	5	5	5	4	4	5	5	1	5	5	1	4	3	3	1	5	1	4	1	4	1	1	1	4	1	4	1	5	1	4
2	4	4	2	5	4	4	4	4	3	3	4	4	3	4	2	4	1	4	3	4	1	3	4	4	1	4	3	4	1	4	1	4	1	4
2	5	5	3	2	2	2	4	4	4	5	4	3	4	4	2	4	3	4	3	5	1	4	3	4	3	2	3	5	3	4	3	4	3	4
3	4	4	1	4	4	2	4	2	4	4	4	1	4	4	2	4	1	4	1	4	1	4	1	4	1	4	1	4	1	4	1	4	1	4
2	2	2	3	2	2	2	2	2	2	2	3	3	2	2	3	2	3	2	3	2	3	3	1	2	1	3	3	3	1	2	3	2	1	3
2	5	5	4	5	5	4	5	5	4	4	5	1	4	5	3	4	3	4	3	5	3	4	1	5	1	5	1	4	1	2	1	4	1	4
1	5	4	1	2	3	3	4	3	4	4	4	3	4	4	1	4	2	2	2	3	2	2	2	2	2	3	2	3	2	2	2	2	1	2
1	5	5	1	4	4	4	4	4	4	5	2	1	4	1	2	4	3	4	3	4	1	5	1	5	1	3	1	4	1	4	1	4	1	2
2	4	5	4	4	4	4	4	4	3	3	3	4	2	4	2	4	3	4	3	4	3	4	4	4	2	4	3	4	2	4	4	4	2	4
1	4	4	1	4	4	4	4	4	4	4	2	1	2	2	1	2	3	3	3	3	3	2	4	4	3	1	1	3	3	1	1	2	1	2
1	4	3	3	5	5	5	5	4	4	4	2	1	4	5	3	4	1	3	1	4	1	4	1	4	1	2	1	4	1	4	1	4	1	4
2	4	5	1	5	5	4	3	4	4	5	4	3	5	5	1	5	3	5	3	4	3	5	3	4	3	5	4	3	3	5	3	5	3	4
1	5	3	3	5	5	4	3	3	2	1	4	3	1	3	3	3	3	3	1	2	1	5	3	5	3	5	3	5	3	5	3	5	3	4
1	4	4	1	4	4	4	4	4	3	4	4	3	4	4	3	4	1	4	1	4	1	4	1	4	1	4	1	4	1	4	1	4	1	4
3	2	2	3	2	2	2	2	4	3	2	1	1	5	4	3	2	1	4	1	5	1	4	3	4	1	2	1	2	1	4	1	4	4	1
5	5	5	1	5	5	5	5	5	5	5	5	1	1	5	5	5	1	5	1	5	1	5	5	5	1	5	1	1	1	5	1	5	1	5

3	5	5	1	5	1	5	5	4	4	3	3	3	5	5	5	3	4	4	4	4	4	2	3	4	1	2	2	4	1	5	3	4	1	4
1	5	5	1	5	5	5	5	5	5	5	5	3	5	5	1	5	4	5	1	5	1	5	1	5	1	5	1	5	1	4	1	5	1	4
1	2	4	1	5	4	4	3	4	2	5	3	3	4	5	2	5	3	5	3	5	3	5	4	4	3	5	3	4	2	4	2	5	2	4
3	4	5	4	4	5	4	5	4	5	5	4	3	3	5	3	4	3	5	1	4	3	5	4	4	3	2	3	4	3	4	1	3	3	3
1	4	4	1	4	4	4	4	4	3	4	5	4	4	4	3	4	3	3	1	4	3	3	3	4	3	4	3	4	1	4	3	4	3	5
1	2	4	3	4	4	4	3	4	3	3	4	3	3	4	3	4	3	5	3	4	3	4	4	4	2	4	3	4	2	4	2	4	2	4
3	4	2	2	5	2	2	5	2	3	3	3	3	2	4	2	3	3	2	3	2	3	3	3	2	3	2	3	2	3	2	3	2	3	2
4	5	5	1	4	4	4	5	4	4	5	4	2	4	4	1	4	1	4	1	5	1	5	1	5	1	4	1	5	1	4	1	5	1	4
1	5	4	3	3	4	2	3	1	1	1	2	2	2	2	5	3	3	5	5	5	1	3	3	3	3	3	3	5	1	1	1	5	1	5
1	5	5	1	5	4	4	5	4	4	4	4	1	5	5	1	5	3	5	1	5	2	5	5	5	2	4	3	3	1	5	1	5	1	5
3	5	5	3	4	4	4	4	4	4	4	4	1	4	4	1	4	3	4	4	4	4	4	4	4	3	4	4	4	3	4	4	4	4	5
2	4	4	4	4	4	4	4	4	3	5	4	3	4	4	4	4	3	4	3	5	3	5	3	5	3	3	3	4	3	5	3	4	3	5
1	5	5	3	4	5	4	5	4	3	4	5	1	5	5	4	4	1	4	1	5	1	5	1	5	1	4	1	5	1	5	1	5	1	5
1	2	2	3	2	4	2	4	2	1	1	4	4	4	4	2	4	1	1	1	4	1	5	4	3	3	5	3	5	4	4	3	4	4	2
2	4	4	1	4	4	4	4	3	3	4	4	1	4	4	1	4	3	5	3	4	3	4	3	4	2	4	2	4	2	4	2	4	2	4
1	5	5	1	5	5	5	5	4	3	5	5	1	5	5	1	5	1	5	1	5	1	5	1	5	1	1	1	5	1	5	1	5	1	5
1	5	4	2	3	4	4	5	4	4	5	5	1	5	4	3	5	3	4	2	5	1	5	3	5	3	5	3	5	3	4	3	5	3	5
1	2	2	1	5	4	4	4	4	4	5	4	1	5	4	1	4	4	4	4	5	4	5	4	5	5	5	4	5	4	4	4	5	4	4
3	4	5	1	4	5	4	4	5	4	4	5	1	4	5	1	4	4	4	4	4	4	4	4	4	3	5	4	4	3	4	4	5	3	4

1	5	5	3	5	5	3	5	4	5	5	4	3	5	5	3	5	1	3	1	5	1	3	1	5	1	3	1	5	1	5	1	5	1	5
2	2	2	3	2	2	2	4	2	4	4	3	3	2	2	3	2	3	2	3	2	3	2	2	2	3	3	3	4	2	2	3	4	3	3
1	3	4	1	3	4	4	5	4	3	1	2	1	5	2	2	3	4	4	4	3	4	4	4	3	4	4	4	3	4	4	4	4	4	4
4	2	3	2	3	2	3	4	2	3	3	4	2	4	3	3	4	3	3	3	4	2	2	2	2	2	3	2	2	3	4	3	1	2	4
1	5	4	3	5	5	4	5	4	3	5	2	1	4	4	3	2	1	3	1	3	1	2	1	2	1	3	1	3	1	3	1	3	1	2
2	2	3	2	5	4	4	3	3	3	4	3	3	2	4	3	5	1	4	1	3	1	4	1	3	1	3	3	4	1	4	2	5	1	2
1	4	4	3	5	4	4	4	4	4	5	4	1	5	4	3	2	1	4	1	3	2	2	2	3	1	1	1	3	1	3	1	4	1	2
1	2	3	3	2	4	4	4	4	2	5	4	5	5	4	3	4	4	5	4	4	4	5	4	4	4	5	5	5	4	4	4	5	4	5
1	4	4	1	4	5	5	5	5	5	5	4	1	4	4	1	4	4	4	2	5	4	3	3	4	4	4	4	4	3	4	4	4	3	4
1	4	3	3	4	4	2	4	3	4	4	3	2	5	4	3	4	3	4	1	5	3	4	3	5	2	3	3	5	2	5	2	5	2	4
1	4	5	3	1	5	4	5	4	5	5	5	1	5	5	3	2	3	5	3	5	4	5	4	5	4	3	4	5	3	5	5	5	3	5
3	3	3	3	3	3	3	4	4	4	4	4	3	5	4	3	4	1	2	2	4	1	3	1	2	1	1	1	2	1	1	1	3	1	3
3	5	2	5	5	5	3	5	3	3	5	3	3	5	5	3	5	5	5	5	5	5	5	5	5	5	5	5	5	5	5	5	5	5	5
2	5	4	1	4	4	4	4	4	4	4	4	4	4	4	4	5	1	2	3	5	1	5	4	2	1	2	1	2	1	2	1	5	1	5
1	4	4	1	3	5	3	1	4	3	4	4	1	4	4	1	3	3	4	3	2	2	5	2	2	2	2	2	3	1	5	2	5	1	5
1	5	5	1	5	5	5	5	5	5	5	5	1	5	5	1	5	4	1	1	5	5	5	5	5	3	5	3	5	3	5	3	5	3	5
1	3	4	3	4	4	4	4	4	3	3	5	3	3	4	3	4	2	4	1	5	3	4	2	5	2	4	2	3	1	5	1	5	1	5
2	2	3	3	3	2	2	2	2	2	1	4	1	1	2	3	3	2	3	2	4	2	4	4	4	3	4	2	4	3	4	4	4	4	4
4	2	2	2	4	2	2	4	3	2	4	3	3	4	4	2	4	3	3	2	3	3	3	4	3	3	3	3	3	3	3	3	4	3	4
4	4	4	4	4	4	4	4	4	4	4	4	4	4	4	4	4	3	4	3	4	3	4	3	4	3	4	3	4	3	4	3	4	3	4

3	5	3	5	5	4	2	5	1	3	1	1	2	1	2	5	4	1	2	1	4	1	4	1	4	1	1	1	4	1	4	1	4	1	3
1	5	5	1	1	5	3	5	3	3	3	4	1	4	5	1	4	1	1	1	3	2	4	1	4	1	1	1	2	1	2	1	1	1	1
1	5	4	2	4	3	3	4	4	3	5	3	1	3	4	1	3	1	3	5	5	1	5	1	5	1	5	3	5	1	5	1	5	1	5
2	4	5	3	5	4	4	5	4	4	3	5	4	1	5	3	5	2	4	3	4	2	4	2	4	3	4	3	4	3	3	3	3	3	3
1	5	5	4	2	4	4	4	5	3	1	5	1	1	3	4	4	3	1	3	2	1	1	2	2	1	1	2	1	1	1	1	5	1	2
1	5	4	3	4	5	4	4	4	1	3	4	1	3	4	4	5	1	1	1	4	1	4	1	4	2	4	2	4	3	2	2	4	1	3
1	2	2	3	2	2	2	5	2	2	5	4	3	5	4	3	4	1	2	1	5	1	4	1	4	1	4	1	4	1	4	1	4	1	4
1	4	3	1	5	3	3	5	3	1	4	3	3	5	4	3	5	1	3	1	5	1	3	1	5	1	3	3	5	1	3	1	5	1	5
1	5	4	1	3	4	4	5	3	4	5	3	3	3	3	1	5	1	3	1	5	1	5	1	5	1	1	1	5	1	5	1	5	1	4
2	5	4	1	3	4	4	5	2	4	5	5	3	5	5	2	2	2	4	2	2	2	2	2	4	1	4	2	3	1	4	2	4	1	4
1	4	2	2	5	4	4	4	4	4	4	4	1	4	4	2	4	1	4	1	5	1	5	1	5	1	1	1	5	1	5	1	5	1	5
3	4	4	2	4	4	4	3	2	3	3	3	2	2	4	1	4	3	4	2	4	2	4	1	4	5	4	3	4	1	4	1	4	1	4
1	5	5	5	2	4	4	4	3	3	1	2	4	5	5	5	2	1	2	2	3	2	2	2	5	5	4	3	3	1	4	2	5	2	2
2	4	4	3	4	4	3	4	4	3	4	3	2	4	4	3	4	1	1	2	4	3	4	3	4	2	1	2	3	1	5	2	5	2	4
1	4	4	3	5	4	4	4	4	3	5	4	3	4	4	3	3	2	4	2	3	2	4	2	4	2	4	2	3	2	4	2	4	2	4
1	4	4	3	4	5	4	5	5	4	5	5	1	5	5	1	5	3	4	4	5	4	4	4	5	3	5	4	5	3	5	4	5	3	4
1	4	4	1	3	4	2	4	2	3	4	4	1	4	4	1	2	1	2	1	1	1	2	1	2	1	1	1	1	1	2	1	2	1	2
1	3	3	2	2	2	3	5	1	3	4	2	1	3	2	5	2	1	2	1	4	1	3	3	2	1	2	1	2	1	2	1	4	1	3
5	4	4	1	5	5	4	4	3	3	5	5	5	5	5	3	5	1	4	1	4	1	5	1	5	1	5	1	5	1	5	1	5	1	5
3	4	5	1	4	4	4	5	4	4	5	5	2	5	5	1	5	3	3	1	4	2	4	3	5	4	3	3	5	2	2	2	4	4	2
1	4	5	5	5	5	5	5	4	3	3	4	1	1	4	1	5	4	5	5	5	3	5	4	4	4	5	4	5	4	4	5	5	4	5
1	4	4	1	4	4	2	4	2	2	2	2	1	2	5	1	2	1	3	1	2	1	4	1	2	1	2	1	2	1	1	1	2	1	2

1	4	2	3	5	2	3	2	2	2	2	2	3	2	5	3	2	1	2	1	2	3	2	1	2	1	2	1	2	1	2	1	2	1	2
1	4	4	1	4	4	4	4	4	4	4	4	3	4	4	1	4	1	3	1	4	1	4	1	4	1	1	4	4	1	4	3	4	1	4
2	2	4	1	4	5	4	4	5	3	5	4	3	5	5	3	4	3	4	2	4	2	4	4	5	3	4	4	5	4	2	4	4	4	4
2	4	2	2	2	2	4	3	3	5	4	3	2	5	4	2	4	4	4	4	4	4	4	4	4	3	3	4	4	3	4	3	5	2	4
2	2	4	3	5	5	4	4	4	3	3	4	1	2	2	2	4	1	4	4	4	1	4	4	4	1	4	1	4	1	4	1	4	1	4
2	5	4	1	5	5	4	5	4	4	4	4	3	4	5	3	4	1	4	1	4	1	4	1	4	1	2	1	4	1	4	1	4	1	3
1	5	4	2	2	4	4	5	4	5	5	3	1	5	5	2	4	1	2	1	4	1	4	1	4	1	2	1	4	1	3	1	5	1	4
1	5	5	2	4	4	3	5	3	3	5	4	1	5	4	3	4	1	1	1	3	1	4	1	4	1	2	1	4	1	2	1	4	1	3
4	4	3	1	2	3	4	3	3	2	1	2	3	2	4	3	2	2	2	1	2	4	3	1	5	1	2	1	4	1	4	1	5	1	5
1	5	5	3	5	5	5	5	4	5	5	5	1	5	5	3	5	3	3	3	3	3	5	1	5	1	1	1	5	1	5	1	5	1	5
3	3	3	3	5	3	3	3	4	2	4	2	4	3	2	1	2	1	2	1	2	2	5	3	2	3	3	2	4	2	2	3	2	2	5
3	4	4	3	4	4	3	4	3	3	4	3	3	4	4	4	4	2	4	4	4	2	4	2	4	2	3	2	4	2	4	2	5	2	3
1	4	3	2	4	4	3	4	2	1	3	3	3	3	3	2	3	1	2	1	4	1	4	1	4	1	3	1	4	1	4	1	4	1	3
1	2	2	3	4	4	3	4	4	4	4	3	3	4	4	3	4	2	4	2	3	2	3	2	4	2	4	3	3	2	3	2	4	2	4
3	5	3	3	4	5	4	4	3	3	4	4	3	3	4	3	4	1	3	2	3	3	3	2	2	1	3	1	3	3	3	3	4	1	3
3	4	4	3	3	4	4	3	1	4	4	1	3	1	4	3	4	3	3	1	2	3	4	3	4	3	1	3	4	3	2	1	4	3	4
2	4	3	2	4	4	4	5	4	4	4	3	3	3	4	2	4	3	5	3	3	3	5	3	5	3	5	3	4	3	5	3	5	3	5
3	4	3	2	3	2	2	5	2	3	3	2	2	4	4	5	4	3	4	2	3	2	4	3	4	1	2	3	3	2	2	3	2	4	3
1	4	4	3	4	4	4	4	4	3	4	4	3	1	2	3	5	2	4	3	5	3	4	3	4	3	4	3	4	3	4	3	4	3	4
2	4	2	3	4	4	4	4	4	4	4	4	2	4	4	2	4	4	4	4	4	4	4	4	4	4	4	4	4	2	4	4	4	4	4
5	5	5	4	5	5	5	5	5	5	4	4	4	4	4	4	4	4	5	3	4	4	4	4	4	1	1	2	4	3	4	3	4	1	1
2	5	4	3	2	2	2	2	2	2	4	2	1	2	2	3	4	3	3	3	4	3	3	2	2	3	1	3	2	3	2	3	2	3	4
2	4	5	4	4	5	4	5	4	4	5	4	1	5	4	4	5	1	3	1	2	1	4	1	4	1	3	1	4	1	4	1	5	1	5

3	5	5	1	5	5	4	3	5	3	4	5	1	2	5	2	2	3	4	4	5	3	4	3	4	3	4	4	5	3	5	3	5	2	4
1	4	2	2	5	5	2	4		4	3	4	3	2	4	2	4	2	4	1	5	1	4	1	4	1	3	1	5	1	4	1	5	1	5
1	5	5	3	5	5	4	5	4	3	5	4	3	4	4	3	4	1	2	2	2	3	5	1	4	1	3	3	4	1	3	1	5	1	3
	5	4	3	5	4	4	5	4	3	3	4	1	4	4	3	4	1	2	3	2	1	2	1	4	4	3	4	3	2	2	1	3	1	1
2	4	4	4	4	4	3	2	4	2	2	2	3	2	2	3	2	3	3	1	3	1	3	1	3	1	2	1	3	1	3	1	2	1	2
1	4	2	2	2	2	3	4	3	1	1	2	2	2	3	3	4	2	4	2	4	2	3	2	4	2	4	2	4	2	4	2	4	2	4
1	4	4	3	4	5	4	4	2	2	2	5	1	2	4	3	5	3	4	2	5	4	4	2	4	3	2	2	2	3	4	2	5	3	4
1	5	5	1	5	4	3	4	4	3	4	4	3	5	5	1	4	1	1	1	4	1	2	1	2	1	1	1	1	1	3	1	2	1	3
1	4	4	4	4	3	3	4	4	3	3	4	1	3	4	4	3	4	1	1	4	1	4	1	4	1	4	1	4	1	4	1	4	1	4
1	5	5	1	4	5	3	3	5	2	4	5	3	3	5	3	4	3	3	3	5	4	5	4	5	5	4	4	4	1	5	3	5	3	5
1	5	5	1	4	5	5	5	4	5	5	4	1	5	5	1	5	1	4	4	4	3	5	3	5	1	4	1	4	1	4	3	5	1	5
1	5	4	1	5	5	5	5	5	5	5	5	1	5	5	1	5	1	1	1	5	5	5	1	5	1	5	1	5	1	5	1	5	1	5
1	5	2	3	5	4	3	5	4	1	5	1	1	5	4	4	4	1	4	1	5	1	5	3	5	1	4	1	3	1	5	1	4	1	3
2	2	2	1	2	2	2	3	2	3	2	2	3	2	2	3	2	3	2	2	2	2	2	2	2	1	2	2	2	1	2	2	2	1	2
1	5	3	2	4	3	3	5	3	3	5	4	1	5	3	2	5	4	5	3	5	3		4	5	3	4	3	5	1	5	1	5	3	5
1	4	5	3	4	5	4	5	4	4	5	4	3	4	5	3	5	4	5	4	5	1	5	1	5	1	3	3	5	3	4	3	5	4	4
1	5	4	1	4	4	5	5	4	4	4	4	1	5	5	1	5	1	4	1	5	1	5	5	5	1	3	4	4	1	4	4	4	1	4
1	2	4	2	3	3	2	4	2	3	5	5	3	5	5	4	4	3	5	1	5	1		1	5	1	5	1	5	1	5	1	5	1	5
4	4	2	2	2	2	2	5	4	4	5	4	1	4	4	4	2	2	5	1	5	3	4	3	5	1	5	3	5	1	3	3	5	3	3
1	5	4	1	3	5	5	5	5	5	5	5	1	5	5	1	5	1	5	5	5	5	5	5	5	1	5	3	5	1	5	5	5	1	5
2	5	5	1	4	5	4	5	4	5	4	5	1	5	5	1	4	3	5	4	5	5	4	1	4	4	4	4	4	3	4	3	4	4	4

2	2	2	3	2	3	5	4	1	2	2	5	3	2	2	2	2	2	2	4	5	5	4	5	5	4	3	1	4	4	3	3	3	3	4
1	5	5	1	5	5	4	4	4	5	3	4	1	2	4	1	5	4	4	4	4	3	4	4	4	1	2	2	4	3	5	4	5	1	4
1	5	3	1	5	5	5	5	3	5	5	3	1	5	5	1	5	5	5	5	5	5	5	2	5	4	1	3	5	2	5	3	3	3	5
1	5	5	1	4	4	4	5	4	4	5	3	1	5	5	1	5	1	1	1	4	1	4	1	4	1	1	1	5	1	4	1	5	1	4
2	5	4	2	5	5	5	4	4	4	5	2	1	5	4	1	5	1	5	1	5	1	5	1	5	1	4	1	5	1	5	1	5	1	5
2	5	4	1	4	5	5	5	5	5	5	5	1	5	5	1	5	5	5	3	5	2	4	4	5	3	4	3	4	4	5	3	5	3	5
1	5	4	3	5	5	3	5	4	2	5	2	1	5	5	2	3	1	4	3	5	2	3	3	5	3	3	3	5	1	2	2	5	1	3
1	5	2	1	5	4	3	5	4	5	5	1	5	5	4	3	4	3	4	1	4	1	4	1	4	1	4	1	5	1	4	1	4	1	4
2	5	4	5	5	5	3	4	4	3	5	4	1	5	5	5	2	1	2	1	4	1	2	1	2	1	2	1	2	1	2	1	2	1	2
5	1	4	2	4	5	5	5	5	4	5	5	3	5	5	1	4	5	5	5	5	3	5	5	5	5	5	5	5	5	5	5	5	5	5
3	5	4	4	4	4	3	5	5	4	5	5	4	5	4	4	5	1	3	1	4	1	4	1	4	1	4	1	4	1	4	1	4	1	5
1	5	4	1	5	5	4	5	4	3	4	4	1	4	4	3	4	3	3	1	2	1	4	1	5	1	1	1	2	1	5	1	5	1	4
2	2	4	2	3	4	3	2	2	2	5	3	3	3	3	4	5	1	4	3	4	3	5	3	5	3	4	3	5	3	4	3	5	3	5
1	5	4	1	5	5	3	5	4	5	5	4	1	5	5	1	4	1	3	1	4	1	5	1	5	1	3	1	3	1	2	1	4	1	3
1	4	4	1	4	4	4	5	3	4	5	3	1	4	4	1	4	1	3	1	4	1	4	1	4	1	2	1	4	1	4	1	4	1	4
2	5	5	1	5	5	4	4	5	3	4	5	1	5	5	1	5	2	4	2	4	2	5	2	5	2	4	2	4	1	4	1	5	1	5
4	4	4	3	3	3	3	3	3	3	4	3	4	3	3	1	4	4	4	3	4	4	4	4	4	4	4	4	1	4	4	4	3	3	4
1	3	4	1	5	4	4	4	4	3	5	4	2	5	4	1	4	2	4	1	3	1	3	3	4	1	4	1	3	1	4	1	4	1	4
1	3	4	3	4	4	2	4	2	2	4	3	3	4	4	3	3	1	2	1	3	1	2	1	4	1	1	1	2	1	2	1	4	1	3
1	5	4	3	4	4	3	5	2	4	5	2	3	5	4	4	3	3	3	1	4	3	4	1	4	1	3	1	4	1	4	1	5	1	4

1	5	2	4	2	2	2	5	2	4	5	4	1	5	5	4	4	1	2	1	5	1	5	1	5	1	2	1	2	1	4	1	5	1	3
1	5	5	1	5	5	5	5	5	4	5	5	1	5	5	1	5	3	4	4	5	4	5	4	5	3	4	4	5	3	4	3	5	4	1
3	5	4	3	4	4	4	4	4	4	4	4	4	4	4	4	4	3	4	1	2	3	2	3	2	1	5	3	2	1	2	1	2	1	2
4	5	4	2	4	4	3	3	3	3	4	4	3	4	3	2	4	3	2	3	4	3	4	3	4	3	4	3	4	3	4	3	4	3	4
1	5	5	3	4	4	4	4	3	3	4	4	1	4	4	3	4	1	2	1	5	1	2	1	5	1	4	1	5	1	4	1	5	1	4
2	4	4	3	4	3	4	4	4	4	4	4	3	3	4	4	4	3	3	3	4	3	4	3	4	3	4	3	3	3	4	3	4	3	4
1	5	5	3	5	5	3	4	4	3	3	4	3	3	4	3	4	1	2	1	4	1	2	1	2	1	2	1	2	1	2	1	2	1	2
3	5	5	4	4	4	3	4	4	2	4	5	3	2	4	2	5	1	5	4	5	3	5	4	5	1	5	2	3	1	5	3	5	3	4
1	5	2	3	2	2	2	4	3	3	3	3	3	2	3	3	2	3	3	1	3	1	1	1	2	1	3	1	3	1	3	1	2	1	2
1	5	5	3	5	5	4	5	5	4	5	4	1	5	5	1	5	4	2	5	5	5	5	5	4	5	5	5	5	5	5	5	5	5	5
3	5	5	4	5	5	3	4	4	3	4	5	3	4	4	2	5	1	3	1	3	1	5	3	5	1	2	1	4	1	5	1	5	1	5
2	4	4	1	4	4	4	4	4	4	5	4	1	5	5	1	5	4	5	4	4	4	5	5	5	3	4	5	4	3	4	4	4	3	5
1	5	5	5	5	5	5	3	5	1	3	5	1	3	5	5	5	1	5	3	5	1	5	1	5	1	2	1	5	1	5	1	5	1	5
1	5	3	3	5	2	4	4	2	4	4	2	1	4	2	1	4	1	3	1	4	1	4	1	4	1	3	1	3	1	4	1	4	1	4
1	5	4	1	5	5	5	5	3	4	5	5	1	5	5	1	5	1	4	1	4	1	5	1	5	1	4	1	1	1	4	1	4	1	4
1	4	4	1	4	4	4	4	4	4	4	5	1	5	5	1	3	1	4	1	4	3	4	2	4	1	3	2	4	1	2	2	5	2	4
2	5	5	3	5	4	4	5	4	5	4	3	1	4	5	3	5	4	4	4	5	4	5	3	5	3	4	3	4	3	4	3	4	3	4
1	5	5	1	4	5	5	5	5	5	5	5	1	5	5	1	4	1	1	1	4	1	2	1	4	1	4	1	5	1	4	1	4	1	3
3	5	4	3	5	5	4	4	4	3	3	1	2	2	3	3	4	3	4	1	2	1	4	1	4	1	3	3	3	1	3	1	3	1	4
2	5	4	3	2	4	3	5	4	3	3	5	3	3	3	3	5	1	2	1	2	1	1	1	4	1	1	1	2	1	3	1	2	1	3
3	2	4	3	3	4	5	4	4	4	5	3	3	4	5	4	5	5	5	5	5	5	5	1	5	5	1	5	5	5	5	1	5	5	5
1	4	4	1	4	4	3	4	3	3	4	4	1	4	4	1	3	1	1	1	2	1	3	1	4	1	1	1	2	1	3	1	2	1	3

1	2	2	1	5	5	4	5	4	3	5	4	3	5	4	1	5	1	3	1	2	1	5	1	1	1	1	1	4	1	5	1	5	1	5
2	4	5	2	4	4	4	5	4	4	3	2	1	1	5	1	3	2	4	3	3	3	4	3	5	2	4	3	4	2	4	2	5	2	4
1	4	4	3	3	4	2	4	3	3	3	4	1	3	4	2	3	1	4	1	4	1	4	1	4	1	3	1	2	1	2	1	4	1	3
3	5	5	4	5	4	3	3	5	3	3	4	1	3	4	5	5	1	4	1	4	1	4	1	4	1	1	1	4	1	4	1	5	1	5
2	2	3	2	2	2	2	2	2	2	2	2	2	2	2	2	3	2	2	2	2	2	2	2	2	2	2	2	2	3	2	2	2	3	2
3	4	4	4	4	4	3	5	3	2	3	2	3	3	4	4	4	3	3	3	3	2	4	3	3	2	4	3	3	2	4	3	4	2	4
2	4	5	3	3	5	4	5	4	4	4	4	3	4	4	4	4	4	4	5	5	4	4	4	4	4	4	4	4	4	4	4	4	4	3
1	5	5	1	3	5	4	5	3	3	5	4	1	5	5	3	5	2	1	1	5	1	5	1	5	1	1	1	5	1	4	1	1	1	4
4	5	5	1	5	5	4	4	4	4	5	5	1	5	5	1	5	4	5	3	4	4	5	3	5	4	5	5	5	4	5	3	5	3	5
3	4	4	4	4	4	4	5	4	5	4	2	2	4	4	2	3	3	5	4	4	3	4	3	4	3	3	3	3	3	4	3	4	3	4
1	4	4	3	4	4	3	2	4	1	5	5	3	5	4	3	4	1	4	1	4	2	5	3	5	1	4	2	4	1	4	1	4	1	4
1	5	3	2	5	5	5	4	4	1	5	3	1	5	3	3	5	3	4	1	1	3	5	2	5	4	4	4	4	3	5	4	5	3	5
2	5	2	1	1	3	3	5	2	2	2	2	3	5	2	3	5	1	2	1	4	1	3	1	5	1	2	1	4	1	4	1	5	1	4
4	4	3	4	5	4	4	3	4	1	2	5	1	2	4	4	5	1	3	1	4	1	4	1	4	1	3	2	3	1	4	1	2	1	4
3	5	5	3	5	5	4	4	4	4	2	2	3	3	3	4	5	1	1	1	4	1	5	1	5	1	3	1	4	1	4	1	5	1	4
3	4	4	3	4	4	3	4	4	4	4	4	3	4	4	3	4	3	4	1	4	1	3	1	4	1	3	1	3	1	4	1	4	1	4
2	2	4	2	2	4	2	2	2	2	1	2	1	2	4	2	2	1	3	1	2	1	1	1	2	1	2	1	3	3	3	3	2	3	2
3	4	3	1	3	4	4	5	4	4	5	4	1	5	4	2	4	4	5	4	5	4	5	4	5	4	3	4	5	4	4	3	5	4	4
1	4	4	1	2	4	2	4	4	4	4	4	1	4	4	1	5	1	2	1	4	1	4	1	4	1	4	1	4	1	4	1	2	1	4
5	3	2	4	3	4	3	5	4	3	3	4	3	5	4	4	4	2	4	2	3	1	4	4	5	1	5	3	2	1	5	1	5	1	5
5	5	4	3	5	4	4	5	5	5	5	5	4	4	4	4	4	1	5	1	4	4	4	3	4	1	4	3	5	3	4	4	5	1	4
3	4	2	3	2	2	3	2	2	2	2	2	3	3	3	3	3	3	3	3	2	3	2	2	2	3	3	3	2	3	2	3	2	3	2

3	3	3	3	5	4	4	3	2	1	3	3	3	3	3	3	3	3	3	3	3	3	5	4	2	3	2	2	3	2	2	4	3	3	5
1	5	5	3	5	5	4	4	4	4	4	5	1	4	5	1	5	4	5	5	5	5	5	5	5	5	5	5	5	4	5	5	5	4	5
1	4	4	4	4	4	4	2	4	4	2	2	2	2	2	2	2		1	1	1	1	2	1	2	1	1	1	2	1	2	1	2	1	2
1	4	2	3	2	3	4	5	4	4	4	3	2	5	3	3	4	3	4	3	4	2	4	2	4	2	5	2	4	2	4	2	4	2	4
1	5	5	1	5	5	4	5	4	3	5	4	2	5	5	1	4	2	5	1	5	2	5	2	5	2	5	2	5	1	5	2	5	1	5
1	5	3	1	5	5	5	5	5	5	5	4	1	5	5	1	5	1	4	1	5	1	1	1	5	1	4	1	4	1	5	1	5	1	1
5	5	5	2	5	5	4	4	5	5	5	4	1	5	5	2	5	5	5	5	5	1	4	5	5	5	5	5	5	5	5	5	5	4	5
2	4	4	3	4	4	4	4	3	2	1	4	1	2	4	2	4	4	4	4	4	4	4	5	5	4	4	4	4	4	4	4	4	4	4
3	5	4	4	5	5	3	5	4	4	5	3	1	5	4	3	5	1	3	1	4	1	5	1	5	1	3	1	3	1	4	1	5	1	4
2	5	4	1	4	4	4	4	3	4	4	4	3	4	4	3	4	3	4	1	5	1	5	1	5	1	4	3	5	1	4	1	5	1	5
1	2	2	3	4	4	2	5	4	4	5	4	3	4	4	2	4	3	5	3	5	3	4	3	2	3	2	3	4	3	4	3	3	3	4
1	5	3	1	5	5	5	5	5	3	5	5	1	5	5	1	5	5	5	5	5	5	5	5	5	5	5	5	5	5	5	5	5	5	5
2	4	5	3	4	4	4	5	4	3	5	5	4	4	5	3	2	2	4	3	4	4	4	2	3	2	3	2	2	2	4	2	5	2	4
5	4	4	4	3	5	5	5	4	5	4	4	3	4	5	4	4	4	4	4	4	3	4	4	4	2	4	1	4	1	4	1	4	1	4
2	2	2	4	4	4	4	4	4	4	4	4	2	4	4	4	4	2	4	4	4	2	4	1	4	1	4	1	4	3	4	2	4	1	4

PG10	Socio-professional categories	Degree	Years after "Baccalauréat"	Marital Situation	Numbers of children	Age of children	Region, city, country
3	4 et 5	6	5	1	0		1
1	2. Artisan, commerçant(e) et chef d'entreprise	6. Diplômes d'études supérieures	3	3. Célibataire	1	1	3. Un pays étranger
1	9. Etudiant(e)	›mes d'études supé	4	3. Célibataire			1. Région Provence Alpes-Côte d'Azur
1	3. Cadres et professions intellectuelles supérieures	ômes d'études supé	5	3. Célibataire			1. Région Provence Alpes-Côte d'Azur
1	3. Cadres et professions intellectuelles supérieures	6. Diplômes d'études supérieures	3	1. Marié(e), pacsé(e), en couple	2	13et 18	1. Région Provence Alpes-Côte d'Azur
3	3. Cadres et professions intellectuelles supérieures	6. Diplômes d'études supérieures	6	1. Marié(e), pacsé(e), en couple	2	2 et 6	1. Région Provence Alpes-Côte d'Azur
1	fonction publique, policier(ère) et militaire, employé(e) administratif	6. Diplômes d'études supérieures	3	3. Célibataire	0	0	2. Autres régions de France
1	3. Cadres et professions intellectuelles supérieures	5. Baccalauréat		1. Marié(e), pacsé(e), en (	2	7 – 10	1. Région Provence Alpes-Côte d'Azur
4	2. Artisan, commerçant(e), et chef d'entreprise	5. Baccalauréat	0	é(e), pacsé(e), en	2	12 – 15	1. Région Provence Alpes-Côte d'Azur
4	3. Cadres et professions intellectuelles supérieures	4. CAP, BEP	0	3. Célibataire	1	8 et demi	1. Région Provence Alpes-Côte d'Azur
4	5	5	0	1	1	23	1
1	3	6		1	2	25 et 29	1
1	fonction publique, policier(ère) et militaire, employé(e) administratif	6. Diplômes d'études supérieures	3	3. Célibataire	0	0	1. Région Provence Alpes-Côte d'Azur
2	2	6	4	1	2	10 et 113	1
1	5	5	0	3	3	7, 8 et 21	1
1	3. Cadres et professions intellectuelles supérieures	›mes d'études supé	6	é(e), pacsé(e), en	1	11	2. Autres régions de France
1	des écoles, instituteur(trice) et assimilé(e)s), technicien(ne), contremaître, agent de maîtrise	›mes d'études supé	4	3. Célibataire	0		1. Région Provence Alpes-Côte d'Azur
1	3. Cadres et professions intellectuelles supérieures	›mes d'études supé	32	é(e), pacsé(e), en	2	16 – 18	2. Autres régions de France

1	2	4		1	1	31	1
1	4. Professions intermédiaires (ex : Professeur(e) des écoles, instituteur(trice) et assimilé(e)s), technicien(ne), contremaître, agent de maîtrise	5. Baccalauréat	0	ié(e), pacsé(e), en	1	2	3. Un pays étranger
3	3	4		1	2	14 et 18	1
1	5	6		1	2	11 et 15	1
2	7. Retraité(e)	4. CAP, BEP		1. Marié(e), pacsé(e), en	2	22 – 25	1. Région Provence Alpes-Côte d'Azur
3	1	6	5	1	1	11	1
2	6	4		2	2	22 et 27	2
1	5	4		1	4	27, 25, 20, 17	1
1	3. Cadres et professions intellectuelles supérieures	5. Baccalauréat	2	2. Séparé(e) / Divorcé(e)	3	37 34 22	3. Un pays étranger
1	3. Cadres et professions intellectuelles supérieures	6. Diplômes d'études supérieures	3	1. Marié(e), pacsé(e), en couple	1	28	2. Autres régions de France
1	fonction publique, policier(ère) et militaire, employé(e) administratif	4. CAP, BEP	0	1. Marié(e), pacsé(e), en couple	1	4 mois	1. Région Provence Alpes-Côte d'Azur
1	6. Ouvrier(ère) (qualifié (e) / non qualifié(e))	5. Baccalauréat	2	3. Célibataire	0	0	1. Région Provence Alpes-Côte d'Azur
1	fonction publique, policier(ère) et militaire, employé(e) administratif	4. CAP, BEP	0	1. Marié(e), pacsé(e), en couple	0	0	1. Région Provence Alpes-Côte d'Azur
3	3	6	4	1	1	13	1
4	3. Cadres et professions intellectuelles supérieures	ômes d'études supér	6	ié(e), pacsé(e), en	3	[illegible] – 11 – 1[illegible]	1. Région Provence Alpes-Côte d'Azur
3	3. Cadres et professions intellectuelles supérieures	ômes d'études supér	9	ié(e), pacsé(e), en	2	1 – 3	2. Autres régions de France
1	3	6	10	1 et 2	3	17, 15, 2	2
1	3. Cadres et professions intellectuelles supérieures	ômes d'études supér	5	ié(e), pacsé(e), en	2	10 – 13	1. Région Provence Alpes-Côte d'Azur
1	3	6	3	3	0		1

3	3	6	3	1	2	23 et 26	1
1	3	4	0	1	2	21 et 27	1
1	3	6	5	1	3	11, 6, 1	1
3	3. Cadres et professions intellectuelles supérieures	ômes d'études supér	6	3. Célibataire	0		1. Région Provence Alpes-Côte d'Azur
3	3	5	0	1	2		2
1	3. Cadres et professions intellectuelles supérieures	6. Diplômes d'études supérieures	5	1. Marié(e), pacsé(e), en couple	3	12, 9, 3	2. Autres régions de France
3	5	5	0	1	2	4 et 8	1
1	6. Ouvrier(ère) (qualifié(e) / non qualifié(e))	4. CAP, BEP	0	é(e), pacsé(e), en	2	) – 10 mc	1. Région Provence Alpes-Côte d'Azur
1	5	4		1	1	8	1
3	3. Cadres et professions intellectuelles supérieures	ômes d'études supér	5	ié(e), pacsé(e), en (	3	11 – 9 – 6	2. Autres régions de France
3	5	6	2	1	2	1	1
3	5	4		1	2	4 et 11	1
1	3	6		2	1	3	1
2	6. Ouvrier(ère) (qualifié (e) / non qualifié(e))	4. CAP, BEP	0	1. Marié(e), pacsé(e), en couple	1	34	1. Région Provence Alpes-Côte d'Azur
2	3. Cadres et professions intellectuelles supérieures	6. Diplômes d'études supérieures	2002	3. Célibataire	0	0	2. Autres régions de France
1	2	5	0	1	1	10	1
3	5	6	2	1	0		1
4	3	6		1	2	21 et 28	1
4	3	6	5	3	0		2

1	5. Employé(e)s : Employé(e) civil et agent de service de la fonction publique, policier(e) et militaire, employé(e) administratif			1. Marié(e), pacsé(e), en (	1	20	2. Autres régions de France
3	6. Ouvrier(ère) (qualifié(e) / non qualifié(e))	5. Baccalauréat	15	é(e), pacsé(e), en	0	0	1. Région Provence Alpes-Côte d'Azur
4	4	6	5	1	0		1
4	2. Artisan, commerçant(e) et chef d'entreprise	6. Diplômes d'études supérieures	2	2. Séparé(e) / Divorcé(e)	2	14, 19	2. Autres régions de France
1	3. Cadres et professions intellectuelles supérieures	6. Diplômes d'études supérieures	5	1. Marié(e), pacsé(e), en couple	2	6 et 8	1. Région Provence Alpes-Côte d'Azur
1	6	6	5	1	2	8 et 10	2
1	3	6	5	1	2	6 et 13	1
4	3	6	5	1	3	11, 18 et 20	2
3	5. Employé(e)s : Employé(e) civil et agent de service de la fonction publique, policier(ère) et militaire, employé(e)	6. Diplômes d'études supérieures	3	1. Marié(e), pacsé(e), en couple	2	5 et 7	2. Autres régions de France
2	7. Retraité(e)	4. CAP, BEP	0	1. Marié(e), pacsé(e), en couple	2	30 et 35	1. Région Provence Alpes-Côte d'Azur
5	9. Etudiant(e)	6. Diplômes d'études supérieures	6	1. Marié(e), pacsé(e), en couple	0	0	1. Région Provence Alpes-Côte d'Azur
1	4	5	0	1	2	22 et 26	1
5	2. Artisan, commerçant(e), et chef d'entreprise	ômes d'études supé	2	é(e), pacsé(e), en	3	[illegible] – 22 – 1	1. Région Provence Alpes-Côte d'Azur
1	5. Employé(e)s : Employé(e) civil et agent de service de la fonction publique, policier(ère) et militaire, employé(e)	6. Diplômes d'études supérieures	2	1. Marié(e), pacsé(e), en couple	2	15 et 2	2. Autres régions de France
3	3. Cadres et professions intellectuelles supérieures	ômes d'études supé	5	é(e), pacsé(e), en	0	0	2. Autres régions de France
2	3. Cadres et professions intellectuelles supérieures	ômes d'études supé	4	é(e), pacsé(e), en	0	0	2. Autres régions de France
1	5	6	3	1	2	13 et ?	1
3	6	4		3			1
1	7. Retraité(e)	5. Baccalauréat	0	1. Marié(e), pacsé(e), en couple	1	40	1. Région Provence Alpes-Côte d'Azur
3	3. Cadres et professions intellectuelles supérieures	ômes d'études supé	5	é(e), pacsé(e), en	3	[illegible] – 27 – 1	1. Région Provence Alpes-Côte d'Azur

1	5	5	0	1	1	22	1
1	instituteur(trice) et assimilé(e)s), technicien(ne), contremaître, agent de maîtrise	6. Diplômes d'études supérieures	5	1. Marié(e), pacsé(e), en couple	2	32 30	2. Autres régions de France
1	fonction publique, policier(ère) et militaire, employé(e) administratif	5. Baccalauréat	2	1. Marié(e), pacsé(e), en couple	2	11 et14	2. Autres régions de France
3	2. Artisan, commerçant(e) et chef d'entreprise	6. Diplômes d'études supérieures	5	2. Séparé(e) / Divorcé(e)	3	14, 17, 18	1. Région Provence Alpes-Côte d'Azur
1	6. Ouvrier(ère) (qualifié (e) / non qualifié(e))	4. CAP, BEP	0	1. Marié(e), pacsé(e), en couple	1	1 ans	1. Région Provence Alpes-Côte d'Azur
2	3. Cadres et professions intellectuelles supérieures	ômes d'études supér	5	3. Célibataire	0		2. Autres régions de France
1	2. Artisan, commerçant(e) et chef d'entreprise	supérieures	3	pacsé(e), en couple	0	0	1. Région Provence Alpes-Côte d'Azur
1	service de la fonction publique, policier(e) et militaire, employé(e) administratif	ımes d'études supé	3	é(e), pacsé(e), en	2	1 – 2	1. Région Provence Alpes-Côte d'Azur
1	fonction publique, policier(ère) et militaire, employé(e) administratif	5. Baccalauréat	0	1. Marié(e), pacsé(e), en couple	2	5-1	2. Autres régions de France
2	2	4		2	4	31 et 37	1
1	5. Employé(e)s : Employé(e) civil et agent de service de la fonction publique, policier(ère) et militaire, employé(e)	4. CAP, BEP	0	1. Marié(e), pacsé(e), en couple	3	2 5 13	2. Autres régions de France
1	2. Artisan, commerçant(e), et chef d'entreprise	4. CAP, BEP	0	ié(e), pacsé(e), en	1	18	1. Région Provence Alpes-Côte d'Azur
2	4. Professions intermédiaires (ex: Professeur(e) des écoles, instituteur(trice) et assimilé(e)s), technicien(ne),	5. Baccalauréat	0	1. Marié(e), pacsé(e), en couple	2	18 mois et 6 ans	2. Autres régions de France
2	4	5	2	1		2	1
2	fonction publique, policier(ère) et militaire, employé(e) administratif	4. CAP, BEP	0	1. Marié(e), pacsé(e), en couple	2	7 ET 3	2. Autres régions de France
3	3. Cadres et professions intellectuelles supérieures	6. Diplômes d'études supérieures	5	1. Marié(e), pacsé(e), en couple	0	0	1. Région Provence Alpes-Côte d'Azur
1	4	6	4	3			2
1	instituteur(trice) et assimilé(e)s), technicien(ne), contremaître, agent de maîtrise	6. Diplômes d'études supérieures	5	1. Marié(e), pacsé(e), en couple	2	15 et 10 ans	1. Région Provence Alpes-Côte d'Azur
1	4	6		1	2	16 et 17	1
2	5	6	3	1	0		1
3	3. Cadres et professions intellectuelles supérieures	6. Diplômes d'études supérieures	7	1. Marié(e), pacsé(e), en couple	0	0	1. Région Provence Alpes-Côte d'Azur
1	4	6	2	2	2	18 et 24	2

1	5	5	3	2	2	37 et 34	1
1	6. Ouvrier(ère) (qualifié(e) / non qualifié(e))	4. CAP, BEP	1. Marié(e), pacsé(e), en (		1	16	1. Région Provence Alpes-Côte d'Azur
3	2	6	3	3	0		1
3	2	6	3	1	2	2 et 4	1
1	7	5	0	1	2	29 et 25	1
1	2	6	2	1	2	12, 18	1
1	3	6	5	1	2	3, 7	2
1	3	6	15	3	0		2
2	5. Employé(e)s : Employé(e) civil et agent de service de la fonction publique, policier(ère) et militaire, employé(e)	6. Diplômes d'études supérieures	3	1. Marié(e), pacsé(e), en couple	3	10, 13, 15	3. Un pays étranger
1	6. Ouvrier(ère) (qualifié(e) / non qualifié(e))	4. CAP, BEP	1. Marié(e), pacsé(e), en (		1	21	1. Région Provence Alpes-Côte d'Azur
1	5	4		2	2	10 et 13	1
2	2	6	0	1	2	5 et 8	1
1	3	6	5	1	2	7 et 4	1
2	6. Ouvrier(ère) (qualifié(e) / non qualifié(e))	ômes d'études supéı	2	3. Célibataire	0		2. Autres régions de France
2	4	6	2	1	2	25 et 26	2
3	fonction publique, policier(ère) et militaire, employé(e) administratif	4. CAP, BEP	0	1. Marié(e), pacsé(e), en couple	1	7	1. Région Provence Alpes-Côte d'Azur
3	4	6	3	3	0		1
3	3. Cadres et professions intellectuelles supérieures	ômes d'études supérieures		2. Séparé(e) / Divorcé	2	5 – 1	1. Région Provence Alpes-Côte d'Azur
3	instituteur(trice) et assimilé(e)s), technicien(ne), contremaître, agent de maîtrise	6. Diplômes d'études supérieures	3	1. Marié(e), pacsé(e), en couple	3	9,11,12	1. Région Provence Alpes-Côte d'Azur
4	3. Cadres et professions intellectuelles supérieures	›mes d'études supé	4	éparé(e) / Divorc	1	11	1. Région Provence Alpes-Côte d'Azur
1	5	5	1	1	1	25 et 27 ans	2
2	5	5	0	1	2	4 et 7	1
1	3. Cadres et professions intellectuelles supérieures	6. Diplômes d'études supérieures	5	1. Marié(e), pacsé(e), en couple	0	0	1. Région Provence Alpes-Côte d'Azur

3	5	2		1	3	25, 21, 18	1
1	6	5	0	1	3	8, 12 et 19	1
1	5	6	3	2	1	17	1
1	6	5	0	1	2		1
3	6	4		1	0		2
4	6	5	0	1	0		1
3	4	6	4	1	2	4 mois et 14 ans	1
1	4. Professions intermédiaires (ex: Professeur(e) des écoles, instituteur(trice) et assimilé(e)s), technicien(ne), contremaître, agent de maîtrise	1. Aucun diplôme	0	1. Marié(e), pacsé(e), en couple	2	21 25	1. Région Provence Alpes-Côte d'Azur
1	4	6	2	1	0		2
3	3	6		1	3	30, 28, 27	2
1	5	6	2	3	2	6, 9	1
1	fonction publique, policier(ère) et militaire, employé(e) administratif	6. Diplômes d'études supérieures	2	1. Marié(e), pacsé(e), en couple	0	0	3. Un pays étranger
1	3. Cadres et professions intellectuelles supérieures	6. Diplômes d'études	13	1. Marié(e), pacsé(e), en	3	24 – 21 –	1. Région Provence Alpes-Côte d'Azur
2	2	1		1	2	8, 10	1
3	3. Cadres et professions intellectuelles supérieures	ômes d'études supé	5	ié(e), pacsé(e), en	3	2 – 7 – 12	1. Région Provence Alpes-Côte d'Azur
3	2. Artisan, commerçant(e) et chef d'entreprise	4. CAP, BEP	0	1. Marié(e), pacsé(e), en couple	2	12 et 8 ans	2. Autres régions de France
1	2. Artisan, commerçant(e) et chef d'entreprise	5. Baccalauréat	0	1. Marié(e), pacsé(e), en couple	2	2 et 4 ans	2. Autres régions de France
1	3	6	8	3	0		1
3	3	6	3	1	1	16	1
1	**kiné**	6	4	1	0		2
2	3	6		1	0		2

4	fonction publique, policier(ère) et militaire, employé(e) administratif	6. Diplômes d'études supérieures	3	1. Marié(e), pacsé(e), en couple	0	0	1. Région Provence Alpes-Côte d'Azur
4	5	5	0	1	2	20 et 21	1
3	1. Agriculteur(trice) exploitant(e)	1. Aucun diplôme	0	3. Célibataire	0	0	1. Région Provence Alpes-Côte d'Azur
1	2. Artisan, commerçant(e) et chef d'entreprise	6. Diplômes d'études supérieures	2	2. Séparé(e) / Divorcé(e)	2	2 et 5	1. Région Provence Alpes-Côte d'Azur
1	3	6	3	1	3	14, 16 et 17	2
3	2. Artisan, commerçant(e) et chef d'entreprise	5. Baccalauréat	0	1. Marié(e), pacsé(e), en couple	2	37 et35 ans	2. Autres régions de France
3	2	5	3	2			1
1	3	6	5	3	0		2
1	3	6	8	1	1	16	1
5	2	6	2	1	2	viennent de naître	2
1	3	6	2	1	2	24 et 22	2
1	3	6	5	1	1	21	1
3	5	4		2	1	22	1
1	2. Artisan, commerçant(e) et chef d'entreprise	6. Diplômes d'études supérieures	2	1. Marié(e), pacsé(e), en couple	1	20	1. Région Provence Alpes-Côte d'Azur
1	3	6	3	1	2	7 et 8	1
1	3. Cadres et professions intellectuelles supérieures	6. Diplômes d'études supérieures	5	1. Marié(e), pacsé(e), en couple	1	16 mois	3. Un pays étranger
4	3. Cadres et professions intellectuelles supérieures	ômes d'études supér	5	ié(e), pacsé(e), en couple			1. Région Provence Alpes-Côte d'Azur
2	3	6	5	1	0		1
1	3	6	3				1
1	5. Employé(e)s : Employé(e) civil et agent de service de la fonction publique, policier(ère) et militaire, employé(e)	5. Baccalauréat	2	3. Célibataire	0	0	1. Région Provence Alpes-Côte d'Azur

1	4	5	3	2	2	16 et 16	2
3	3. Cadres et professions intellectuelles supérieures	6. Diplômes d'études supérieures	5	1. Marié(e), pacsé(e), en couple	0	0	1. Région Provence Alpes-Côte d'Azur
1	4	6	3	3	0	0	1
3	5	6		2	2	7 et 10	2
1	2. Artisan, commerçant(e) et chef d'entreprise	6. Diplômes d'études supérieures	4	1. Marié(e), pacsé(e), en couple	2	9 , 10	1. Région Provence Alpes-Côte d'Azur
3	5. Employé(e)s : Employé(e) civil et agent de service de la fonction publique, policier(ère) et militaire, employé(e) administratif	4. CAP, BEP	0	3. Célibataire	3	9 20 24	1. Région Provence Alpes-Côte d'Azur
1	3	6	5	1	0		2
1	3	6	5	1	2	9 et 11 ans	2
1	2	4		1	2	26 et 29	2
5	1. Agriculteur(trice) exploitant(e)	2. Brevet des collèges	0	ié(e), pacsé(e), en [illegible]	0		1. Région Provence Alpes-Côte d'Azur
1	2	4		1	1	31	1
3	3	6	5	3	0		1
1	5	5	3	1	3	4, 9 et 11	2
1	3. Cadres et professions intellectuelles supérieures	6. Diplômes d'études supérieures	5	1. Marié(e), pacsé(e), en couple	2	6 ET 8	2. Autres régions de France
1	3	6	2	3	0		2
2	3. Cadres et professions intellectuelles supérieures	[illegible]ômes d'études supé[illegible]	7	[illegible]é(e), pacsé(e), en [illegible]	3	[illegible] – 34 – [illegible]	3. Un pays étranger
3	2. Artisan, commerçant(e) et chef d'entreprise	2. Brevet des collèges	0	1. Marié(e), pacsé(e), en couple	2	17. 13	2. Autres régions de France
1	3. Cadres et professions intellectuelles supérieures	6. Diplômes d'études supérieures	6	1. Marié(e), pacsé(e), en couple	3	2,4 et 6 ans	2. Autres régions de France
4	2. Artisan, commerçant(e) et chef d'entreprise	4. CAP, BEP	0	2. Séparé(e) / Divorcé(e)	4	12 14 19 22	2. Autres régions de France
1	6. Ouvrier(ère) (qualifié (e) / non qualifié(e))	6. Diplômes d'études supérieures	2	1. Marié(e), pacsé(e), en couple	1	2	1. Région Provence Alpes-Côte d'Azur
5	9	5	0	3	0		1
1	4	6	2	1	2	14 et 17	1

1	3	6	5	1	0		1
2	5	6	3	1	0		2
1	4	6		3			1
1	6. Ouvrier(ère) (qualifié(e) / non qualifié(e))	4. CAP, BEP		3. Célibataire	3	30 – 20	1. Région Provence Alpes-Côte d'Azur
2	3. Cadres et professions intellectuelles supérieures	5. Baccalauréat		1. Marié(e), pacsé(e), en	2		1. Région Provence Alpes-Côte d'Azur
3	3. Cadres et professions intellectuelles supérieures	6. Diplômes d'études supérieures	12	1. Marié(e), pacsé(e), en couple	2	6 et 4	1. Région Provence Alpes-Côte d'Azur
4	6. Ouvrier(ère) (qualifié (e) / non qualifié(e))	6. Diplômes d'études supérieures	2	1. Marié(e), pacsé(e), en couple	2	16 et 14	2. Autres régions de France
1	5	6	3	1	1	3	1
3	3	6	4	2	1	17	2
3	2. Artisan, commerçant(e) et chef d'entreprise	5. Baccalauréat	0	1. Marié(e), pacsé(e), en couple	0	0	1. Région Provence Alpes-Côte d'Azur
1	3	6	11	1	2	3 et 5	1
3	3	6	7	1	0		1
1	4	5	1	1	3	5, 8, 10	2
1	3. Cadres et professions intellectuelles supérieures	6. Diplômes d'études supérieures	5	2. Séparé(e) / Divorcé(e)	2	15, 9	1. Région Provence Alpes-Côte d'Azur
1	6. Ouvrier(ère) (qualifié(e) / non qualifié(e))	4. CAP, BEP		1. Marié(e), pacsé(e), en	1	22	1. Région Provence Alpes-Côte d'Azur
1	6	5	0	1	0		2
2	6	4		1	3	13, 18, 21	2
4	3	6	6	3	0		1
1	5	5	0	1	2	8, 15	2
1	de la fonction publique, policier(e) et militaire, employé(e) administratif	5. Baccalauréat		1. Marié(e), pacsé(e), en	2	6 – 3	1. Région Provence Alpes-Côte d'Azur
1	fonction publique, policier(ère) et militaire, employé(e) administratif	6. Diplômes d'études supérieures	3	1. Marié(e), pacsé(e), en couple	2	2 5	1. Région Provence Alpes-Côte d'Azur
3	fonction publique, policier(ère) et militaire, employé(e) administratif	5. Baccalauréat	2	1. Marié(e), pacsé(e), en couple	2	3 - 5	2. Autres régions de France

2	3	6	6	3	0		2
4	3	6	4	1	3	23, 21 et 21	1
1	1	4			2	35 et 30	2
2	3	6	2	1	1	5	1
1	3. Cadres et professions intellectuelles supérieures	6. Diplômes d'études supérieures	5	1. Marié(e), pacsé(e), en couple	2	3 mois	3. Un pays étranger
1	3. Cadres et professions intellectuelles supérieures	6. Diplômes d'études supérieures	5	1. Marié(e), pacsé(e), en couple	2	5 mois et 3 ans	1. Région Provence Alpes-Côte d'Azur
5	3. Cadres et professions intellectuelles supérieures	ômes d'études supé	4	3. Célibataire	1	29	2. Autres régions de France
4	fonction publique, policier(ère) et militaire, employé(e)	5. Baccalauréat	2	pacsé(e), en couple	2	11 et 14	2. Autres régions de France
1	service de la fonction publique, policier(e) et militaire, employé(e) administratif	ômes d'études supé	5	é(e), pacsé(e), en	2	27 – 18	1. Région Provence Alpes-Côte d'Azur
1	2. Artisan, commerçant(e), et chef d'entreprise	ômes d'études supé	2	3. Célibataire			2. Autres régions de France
3	3	6	4	1	0		2
5	7. Retraité(e)	5. Baccalauréat	2	1. Marié(e), pacsé(e), en couple	2	32 35	2. Autres régions de France
2	8. Personne sans activité professionnelle	ômes d'études supé	5	éparé(e) / Divorcé	2	10 – 12	2. Autres régions de France
1	2	5	0	3	1	17	1
1		6	5	1	3	24, 26 et 30	1
